# DNA methylation arrays as surrogate measures of cell mixture distribution

**DOI:** 10.1186/1471-2105-13-86

**Published:** 2012-05-08

**Authors:** Eugene Andres Houseman, William P Accomando, Devin C Koestler, Brock C Christensen, Carmen J Marsit, Heather H Nelson, John K Wiencke, Karl T Kelsey

**Affiliations:** 1 College of Public Health and Human Sciences, Oregon State University, Corvallis, OR 97331, USA; 2 Department of Pathology and Laboratory Medicine, Brown University, Providence, RI 02912, USA; 3 Section of Biostatistics and Epidemiology, Dartmouth Medical School, Hanover, NH 03755, USA; 4 Department of Epidemiology, University of Minnesota, Minneapolis, MN 55455, USA; 5 Department of Neurological Surgery, University of California San Francisco, San Francisco, CA 94158, USA; 6 Department of Epidemiology, Brown University, Providence, RI 02912, USA

## Abstract

**Background:**

There has been a long-standing need in biomedical research for a method that quantifies the normally mixed composition of leukocytes beyond what is possible by simple histological or flow cytometric assessments. The latter is restricted by the labile nature of protein epitopes, requirements for cell processing, and timely cell analysis. In a diverse array of diseases and following numerous immune-toxic exposures, leukocyte composition will critically inform the underlying immuno-biology to most chronic medical conditions. Emerging research demonstrates that DNA methylation is responsible for cellular differentiation, and when measured in whole peripheral blood, serves to distinguish cancer cases from controls.

**Results:**

Here we present a method, similar to regression calibration, for inferring changes in the distribution of white blood cells between different subpopulations (e.g. cases and controls) using DNA methylation signatures, in combination with a previously obtained external validation set consisting of signatures from purified leukocyte samples. We validate the fundamental idea in a cell mixture reconstruction experiment, then demonstrate our method on DNA methylation data sets from several studies, including data from a Head and Neck Squamous Cell Carcinoma (HNSCC) study and an ovarian cancer study. Our method produces results consistent with prior biological findings, thereby validating the approach.

**Conclusions:**

Our method, in combination with an appropriate external validation set, promises new opportunities for large-scale immunological studies of both disease states and noxious exposures.

## Background

The biology of the development of any multisystem life form is fundamentally grounded in systematic cellular differentiation. This is essentially defined by lineage commitment of cells whose origin can be traced to a pluripotent progenitor and is marked by mitotically heritable epigenetic changes that reflect complex transcriptional programming of gene expression within the individual cell [1-3]. One such epigenetic mark is DNA methylation, which is tightly associated with alterations in the nucleosome DNA scaffold (and hence chromatin) that is responsible for coordination of gene expression in individual cells [1-3]. It is now appreciated that differentially methylated DNA regions (DMRs) distinguish cell lineages with high sensitivity and specificity [4] and considerable research is now underway to delineate precise DMRs that define and specify a particular cell lineage. The most developed understanding of epigenetic markers of lineage commitment to date is perhaps that of immune cell subclasses defined by populations of distinct circulating blood cells [5,6].

Pluripotent hematopoietic stem cells residing in the bone marrow continually give rise to the entire hierarchy of blood cell subclasses through a developmental process known as hematopoiesis. Leukocytes, commonly called white blood cells, are critical in the host response to pathogens and foreign antigens and are divided into two compartments, the myeloid lineage and lymphoid lineage (also called lymphocytes). The composition of leukocyte populations is well known to reflect disease states and toxicant exposures and can be altered by signaling cascades that prompt migration of whole classes of cells into or out of tissues. Several DMRs that serve as reliable biomarkers of individual human white blood cell types have already been identified [5,6]. Individual assays identifying cell-specific DMRs have proven useful for quantifying individual cell types in human tissues and peripheral blood. However, these assays are limited to detecting the relative proportion of one individual cell type compared with all others. On the other hand, simultaneous quantification of fluctuation in overall lymphocyte population composition can be accomplished only by using methods based on flow cytometry, which require large volumes of fresh blood and involve laborious antibody tagging. Hence, an approach that allows for the simultaneous quantification of the entire distribution of cell types, using an array of biomarkers based on generally available technology, would be considerably more informative, especially in studies of human disease and exposures.

In some instances, it is generally the overall balance of leukocyte subclasses in circulation or tissue that most prominently influences pathogenesis. For example, although incipient cancer cells are recognized and eliminated by cytotoxic T-cells (CTLs) and natural killer (NK) cells, tumorigenesis is also promoted by certain other inflammatory cells, including B-lymphocytes, mast cells, neutrophils, regulatory T-cells (Tregs), and numerous others. All of these cells have been shown to promote angiogenesis, tumor cell proliferation, tissue invasion and metastasis [7,8]. Likewise, while higher levels of NK cells and CTLs circulating in the blood and residing in adipose tissues are associated with lower incidence of metabolic diseases such as type II diabetes [9], higher levels of M1 macrophages in adipose tissue can induce inflammation and insulin resistance [10]. These examples illustrate incredible potential for methods of quantifying the composition of lymphocyte populations to critically inform the underlying immuno-biology of disease states as well as the immune response to almost all chronic medical conditions. In addition, they offer great potential for predicting therapeutic outcomes [11].

Here we employ the concept of DMRs as markers of immune cell identity using a high density methylation platform, and propose a set of analytical tools for estimating the proportions of immune cells in unfractionated whole blood that does not require fresh cells. The backbone of the approach is the DNA methylation signature of each of the principal immune components of whole blood (B cells, granulocytes, monocytes, NK cells, and T cells subsets). We essentially seek a form of regression calibration, where we consider a methylation signature to be a high-dimensional multivariate surrogate for the distribution of white blood cells. In turn, this distribution is of interest for predicting or modeling disease states. As a surrogate, the DNA methylation signature is assumed to be a highly correlated, yet imperfect, measure of leukocyte distribution, and thus fits into the framework of measurement error models, where the use of a noisy surrogate marker to investigate an association with a disease outcome of interest results in biased estimates, unless internal or external validation data can be obtained to “calibrate” the model and correct the bias [12]. However, in this case, the problem is complicated by the extremely high dimension of the surrogate, so we propose an alternative to the traditional regression-calibration procedure that circumvents these complications but still allows us to extract the desired biological information.

We note that since we began this work, a small number of authors have published similar deconvolution algorithms using gene expression data [13-15]. The techniques are similar to the quadratic programming method we describe below in Methods for deconvolving a single sample, but none comprehensively addresses statistical properties or employs data from DNA methylation.

## Methods

In this section we describe our proposed statistical methods, the data sets used to demonstrate their utility, and finally the design of simulation studies we have conducted to investigate statistical properties of our proposed algorithms.

### Statistical methods

Let **Y**_0*h*_ be an *m *× 1 vector of methylation assay values, e.g. average beta values from an Infinium bead-array product corresponding to a purified blood sample consisting of a homogenous cellular population (e.g. monocytes or granulocytes), with the qualitative characterization of cell type (among *d*_0_ such types) indicated by a *d*_0_ × 1 covariate vector **w**_*h*_. Here, *h*∈{1,…,*n*_0_}, where *n*_0_ is the number of specimens and the *m* individual values correspond to CpG sites on a DNA methylation microarray, possibly pre-selected to correspond to putative DMRs for distinguishing different cellular types. Correspondingly, let **Y**_1*i*_ be an *m *× 1 vector of methylation assay values for the same CpG sites (in the same order) as **Y**_0*h*_, but corresponding to a heterogeneous mixture of cells (e.g. peripheral whole blood) from a human subject. Here, *i*∈{1,…,*n*_1_}, *n*_1_ is the number of target specimens, and **z**_1*i *_is a *d*_1_×1 covariate vector representing phenotypes or exposures corresponding to the subject, e.g. *d*_1_ = 2 for a simple case/control study without confounders. Our goal is to understand the associations between **Y**_1*i*_ and **z**_1*i*_ in terms of associations between **Y**_0*h *_and **w**_0*h*_, i.e. to infer changes in mixtures of cell types associated with phenotypes or exposures, using DNA methylation as a surrogate measure of cell mixture. Thus, we have two data sets, *S*_0_ = {(**Y**_01_,**w**_1_),…,(**Y**_0*n*__0_,**w**_*n*__0_)}, the set of data from “purified” cell samples effectively representing external validation or gold-standard data, and *S*_1_ = {(**Y**_11_,**z**_1_),…,(**Y**_1*n*__1_,**z**_*n*__1_)}, representing surrogate data collected from a target population. To this end, we posit the following linear models:

(1) $$\begin{array}{l} Y_{0h}=B_{0}w_{0h}+e_{0h}\\ Y_{1i}=B_{1}z_{1i}+e_{1i}, \end{array}$$

where **B**_0_ and **B**_1_ are, respectively, *m *×* d*_0_ and *m *×* d*_1_ matrices and **e**_0_ and **e**_1_ are error vectors. For simplicity we assume a one-way ANOVA parameterization for **w**, though in the Additional file 1 we describe slight generalizations to account for design complications met in practice. We also assume a reasonable regression parameterization for **z**, including an intercept, and for convenience, denote the first column of **B**_0_ as *μ*_1_, the *m *× 1 intercept. The error vectors **e**_0_ and **e**_1_ may reflect independence among arrays *h* and *i*, or else may have more complex random effects structure accounting for technical effects or biological replication; however, their substructures are incidental to this analysis, with the exception of the fine details of the bootstrap procedure proposed below.

To implement a surrogacy relation, we propose the following linking regression model:

(2) $$B_{1}=1_{m}\gamma_{0}^{T}+B_{0}\Gamma +U,$$

where *Γ* is a *d*_0_ ×* d*_1_ matrix that summarizes associations between the rows of **B**_0*j*_ and **B**_1*i*_ and **U** is a matrix of errors. Substituting equation (2) into (1), writing **B**_0_ = (**b**_01_,…,**b**_0*d*__0_) explicitly in terms of its columns and writing $\Gamma^{T}=(\gamma_{1},\ldots ,\gamma_{d_{0}})$, it follows that

(3) $$Y_{1i}=\overset{d_{0}}{\underset{l=0}{\sum }}b_{0l}(\gamma_{l}^{T}z_{1i})+(1_{m}\gamma_{0}^{T}+U)z_{1i}+e_{1i}.$$

To impart a biological interpretation, we assume that the DNA assayed in *S*_1_ arises as a mixture of DNA from cell types profiled in *S*_0_, with mixture coefficients whose population averages, conditional on **z**, are $\left\{\omega_{1}^{\left(z\right)},\ldots ,\omega_{d_{0}}^{\left(z\right)}\right\}$, so that

(4) $$E(Y_{1i}|z_{1i}=z)=\xi^{\left(z\right)}+\overset{d_{0}}{\underset{l=1}{\sum }}b_{0l}\omega_{l}^{\left(z\right)},$$

where the *m *× 1 vector *ξ*^(**z**)^ represents cell types excluded from consideration among the purified samples in *S*_0_, or else non-cell-specific methylation, including alterations at the molecular level in the maintanence of DNA methylation patterns themselves (possibly exposure related, age, or disease related). It follows from (3) and (4) that the mixture coefficients are recoverable from *Γ*, $\omega_{l}^{\left(z\right)}=\gamma_{l}^{T}z_{1i}$, provided *ξ*^(**z**)^ is orthogonal to the column space of **B**_0_. As we discuss in detail in the Additional file 1, bias can arise if differences in *ξ*^(**z**)^ between distinct values of **z** have nonzero projection onto the column space of **B**_0_, although the magnitude of anticipated biases can be assessed through sensitivity analysis.

It is possible to assign interpretations to the components of variation in (3). Let *SS*_*o*_ represents overall variability in **Y**_1*i*_, i.e. $SS_{o}=\overset{n_{1}}{\underset{i=1}{\sum }}∥Y_{1i}-{\bar{\mu }}_{1}{∥}^{2}$, where ${\bar{\mu }}_{1}=E(Y_{1i})$. From multivariate probability theory it is straightforward to show that *SS*_*o *_=* SS*_*e*_ + *SS*_*v*_ + *SS*_*u*_, where $SS_{e}=\overset{n_{1}}{\underset{i=1}{\sum }}∥e_{1i}{∥}^{2}$, $SS_{v}=\overset{n_{1}}{\underset{i=1}{\sum }}{\left(z_{1i}-{\bar{z}}_{1}\right)}^{T}\Gamma^{T}B_{0}^{T}B_{0}\Gamma (z_{1i}-{\bar{z}}_{1})$, and $SS_{u}=\overset{n_{1}}{\underset{i=1}{\sum }}\{{\left(z_{1i}-{\bar{z}}_{1}\right)}^{T}U^{T}U(z_{1i}-{\bar{z}}_{1})+m{\left(z_{1i}-{\bar{z}}_{1}\right)}^{T}\gamma_{0}\gamma_{0}^{T}(z_{1i}-{\bar{z}}_{1})\}$. *SS*_*e*_ measures variation unexplained by the covariates **z**_1*i*_, presumed to represent a combination of technical noise and unsystematic biological heterogeneity. *SS*_*v*_ measures variability explained by mixtures of profiles in the set *S*_0_, while *SS*_*u*_ measures variability in systematic biological heterogeneity that nevertheless remains unexplained by mixtures of profiles in *S*_0_, presumably due to some process other than differences in mixtures of cell types. Thus we propose two partial coefficient of determination measures: $R_{1,0}^{2}=SS_{v}/SS_{o}$, which represents the proportion of total variation in *S*_1_ explained by *S*_0_, and $R_{1,1}^{2}=SS_{v}/(SS_{o}-SS_{e})$, which represents the proportion of systematic variation in *S*_1_ explained by *S*_0_. Note that $R_{1,1}^{2}$ is poorly defined when *SS*_*o*_≈*SS*_*e*_.

Estimation procedes by applying an appropriate linear model, e.g. ordinary least squares, linear mixed effects models [16], limma [17], or surrogate variable analysis [18,19], to obtain estimates ${\hat{B}}_{0}$ and ${\hat{B}}_{1}$. Estimates of *γ*_0_ and *Γ* are then obtained by projecting ${\hat{B}}_{1}$ onto the column space of ${\tilde{B}}_{0}=(1_{m},B_{0})$, as described in detail in the Additional file 1. Standard errors can be obtained in one of three ways. The simplest estimator, *SE*_0_, is the “naive” estimator from simple least-squares theory, ignoring the fact that ${\hat{B}}_{0}$ and ${\hat{B}}_{1}$ are estimates, i.e. potentially variable. To account for variation in estimating ${\hat{B}}_{1}$, a simple alternative is to use a nonparametric bootstrap procedure. For each bootstrap iteration *t*, we sample with replacement from *S*_1_ (or sample errors in a manner consistent with a hierarchical experimental design) to obtain $S_{1}^{\left(t\right)}$, producing bootstrap estimates ${\hat{B}}_{1}^{\left(t\right)}$ from which “single-bootstrap” standard errors *SE*_1_ are computed. Finally, it is possible to account for variation in estimating _**B**0_by also bootstrapping *S*_0_; because of potentially small sample sizes *n*_0_, we propose using a parametric bootstrap. A“double-bootstrap” standard error estimator, *SE*_2_, is computed from these two sets of bootstraps. The double-bootstrap has the additional benefit over the single-bootstrap, in that it can be used to assess bias due to measurement error (variability) in ${\hat{B}}_{0}$. Estimation details are provided in the Additional file 1, as are the results of simulation studies.

Beyond bias due to measurement error, which is easily corrected using the double-bootstrap procedure, there are additional sources of potential bias. For example, consider a univariate *z*_1*i*_ representing case/control status, where $\delta \equiv \xi^{\left(1\right)}-\xi^{\left(0\right)}=B_{0}\alpha$ for some *d*_0_ × 1 vector *α *≠** 0**; i.e. *δ* is the mean difference in DNA methylation between a case and control, contributed by cell mixtures that remain uncharacterized or non-cell-specific methylation. In such a situation, there will be a bias equal to *α* in estimating the mixture differences. The Additional file 1 provides a detailed analysis of such biases, and proposes a sensitivity analysis procedure for assessing the magnitude of possible bias in a given data set.

While the focus of this paper is analysis of population data, it is possible to use *S*_0_ to predict distribution of leukocytes in a single sample having DNA methylation profile **Y**^∗^. Equating the intercept term of **B**_1_ in (1) with **Y**^∗^ and applying (2), we obtain mixing proportion estimates $\Gamma^{*}={\left({\tilde{B}}_{0}^{T}{\tilde{B}}_{0}\right)}^{-1}{\tilde{B}}_{0}^{T}Y^{*}$. Estimates can be further refined with the use of quadratic programming techniques [20], restricting the components of *Γ*^∗^,*γ*_*l*_^∗^ ≥ 0, in minimizing $∥Y^{*}-{\tilde{B}}_{0}\Gamma^{*}{∥}^{2}$ with respect to *Γ*^∗^. Such individual projections of methylation profiles on the column space spanned by *S*_0_ facilitate the application of the fundamental ideas proposed above to individual, clinically-based diagnostic procedures. Note, however, that DNA methylation arrays are typically focused on the comparison of methylated to unmethylated CpG dinucleotides, not quantifying actual amounts of DNA. Therefore, information on cell mixtures from DNA methylation is limited to distributions, not actual counts, as one might obtain from flow cytometry. Finally, we remark that it is possible to model **z**_1*i*_ directly as a function of mixture coefficients *Γ*^∗^ obtained individually via the constraint *γ*_*l*_^∗^ ≥ 0, but the inferential implications are less clear, and we view the proposed approach for populations as more statistically robust.

### Implementation

We describe several examples using existing methylation data sets as benchmarks for validating the proposed method, in order to demonstrate its clinical or epidemiological utility. First we describe the validation data set *S*_0_ used in all examples. Next we describe a laboratory reconstruction experiment, which validates our fundamental proposition that DNA methylation retains substantial information about cell mixtures. Finally we describe the results of applying our methodology to several different target data sets *S*_1_. For the head and neck cancer and ovarian cancer data sets, from which bead chip data were available, a linear mixed effects model with a random intercept for bead chip was used to estimate the corresponding row of **B**_1_. For the remaining data sets, no bead chip data were available; consequently, ordinary least squares was used. 250 bootstrap iterations were used for each example and each of the two bootstrap methods of standard error estimation.

#### Validation data

**Figure 1:**
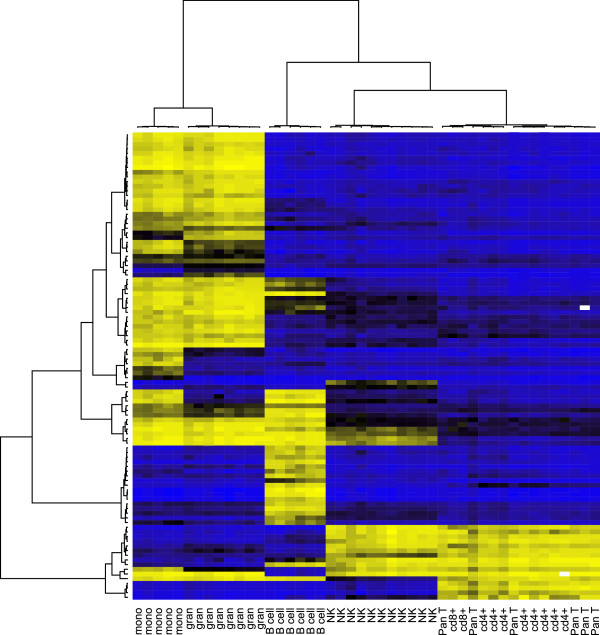
**Clustering heatmap for external validation white blood cell data (***S*_*0*_**).** Yellow = unmethylated (*Y*_*hj *_= 0), black = partially methylated (*Y*_*hj *_= 0.5), blue = methylated (*Y*_*hj *_= 1).

All data analyses involve DNA methylation data obtained by the Infinium HumanMethylation27 Beadchip Microarrays from Illumina, Inc. (San Diego, CA). We used a subset of *m *= 100 CpG sites on the array, selected as described below. In all of our examples, *S*_0_ consisted of 46 white blood cell samples, de-identified specimens that were not subject to human subjects review by an institutional review board (IRB). The sorted, normal, human, peripheral blood leukocyte subtypes were purchased from AllCells^*Ⓡ*^, LLC (Emeryville, CA) and were isolated from whole blood using a combination of negative and positive selection with highly specific cell surface antibodies conjugated to magnetic beads; materials and protocols were obtained from Miltenyi Biotec, Inc. (Auburn, CA). These 46 samples are summarized in Table 1 and depicted by the clustering heatmap in Figure 1. Note that T lymphocytes that express CD4 or CD8 constitute over 95% of the T cell class, and that the pan-T cell type was further refined to CD4+, CD8+, and “other” Pan-T cells subtypes. In summary, the covariate vector **w**_*h*_consisted of indicators for five cell types and another two indicators for CD4+ and CD8+ T cell subtypes. A generalization of the one-way ANOVA parameterization assumed above for **w**_*h*_, described in the Additional file 1, was necessary to account for the ambiguous status of some Pan-T cells. For each CpG site, a linear mixed effects model with a random intercept for bead chip was used to estimate **B**_0_; 27 additional whole blood control samples (replicates from the same individual) were used to assist in estimating chip effects, since otherwise the data set would have been sufficiently sparse to risk confounding between cell type and chip. These “array controls” were indicated with an additional term in **w**_0*h*_. For each CpG site, a linear mixed effects model with a random intercept for bead chip was used to estimate the corresponding row of **B**_0_and **B**_1_. From *S*_0_, *F* statistics (described in the Additional file 1) were computed and used to order each of the 26,486 autosomal CpGs by decreasing level of informativeness with respect to blood cell types. As described in the Additional file 1, we determined that maximum informativeness was provided by the top *m *= 100 − 300 CpG sites, with *m *> 300 reflecting diminishing returns from adding additional CpGs. Therefore, we chose a moderately low value in this range, *m *= 100, consistent with the size of a small custom microarray chip.

**Table 1:** Sorted white blood cells in *S*_0_

**Short name**	**Description**	**Number**
B cells	CD19+ B-lymphocytes	6
Granulocytes	CD15+ granulocytes	8
Monocytes	CD14+ monocytes	5
NK	CD56+ Natural Killer (NK) cells	11
T cells (CD4+)^1,2^	CD3+CD4+ T-lymphocytes	8
T cells (CD8+)^1,3^	CD3+CD8+ T-lymphocytes	2
T cells (NKT)^1^	CD3+CD56+ natural killer	1
T cells (other)^1^	CD3+ T-lymphocytes	5

^1^Considered as a member of the “pan-T-cell” group.

^2^Pan-T-cell further refined as also belonging to the “CD4+” group.

^3^Pan-T-cell further refined as also belonging to the “CD8+” group.

#### Cell mixture experiment

Proof of the utility of the proposed methods in predicting leukocyte distributions for individual samples requires extensive, detailed reconstruction experiments beyond the scope of the present paper. However, to provide evidence that such experiments are worthwhile and show promise of positive results, we conducted a simple experiment involving six known mixtures of monocytes and B cells and six known mixtures of granulocytes and T cells. The results of this experiment are described below in Results.

#### Head and neck cancer

**Figure 2:**
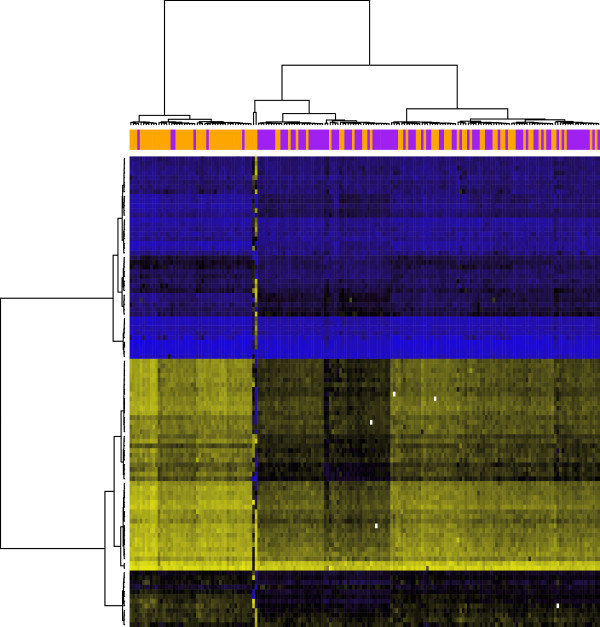
**Clustering heatmap for target HNSCC data (***S*_1_**).** Yellow = unmethylated $\left(Y_{\text{ij}}=0\right)$, black = partially methylated $\left(Y_{\text{ij}}=0.5\right)$, blue = methylated $\left(Y_{\text{ij}}=1\right)$. The annotation track above the heatmap indicates case-control status (orange = case, purple = control).

Our first target data set *S*_1_ consisted of arrays applied to whole blood specimens collected in a random subset of individuals involved in an ongoing population-based case-control study [21] of head and neck cancer (HNSCC): 92 cases and 92 age and sex matched controls. The study was approved by Brown University IRB, protocol #0707992334. Blood was drawn at enrollment (prior to treatment in 85% of the cases). Mean age among the subjects arrayed in this study was 60 years, and there were 56 females and 128 males, consistent with the higher incidence of the disease in men. Thus, the covariate vector **z**consisted of an indicator for case/control status, an indiator for male sex, and age (in decades) centered at the mean. The clustering heatmap in Figure 2 depicts the raw DNA methylation data in *S*_1_.

#### Ovarian cancer

We next applied our method to an ovarian cancer data set [22]. DNA methylation data for blood samples are available from Gene Expression Omnibus (GEO, http://www.ncbi.nlm.nih.gov/geo/, Accession number GSE19711). We used only those cases having blood drawn pre-treatment. After removing 4 arrays with a preponderance of missing values, the data set consisted of 272 controls and 129 cases having blood drawn prior to treatment. A clustering heatmap displaying the DNA methylation data appears in the Additional file 1. In this analysis, **z**consisted of case-control status, age (categorized in 5-year increments), and 2 bisulfite conversion efficiency measures.

#### Down syndrome

We also applied our method to a trisomy 21 (Down syndrome) data set [23] consisting of 29 total peripheral blood leukocyte samples from Down syndrome cases and 21 controls, as well as 6 T cell samples from cases and 4 T cell samples from controls (GEO Accession number GSE25395). Because of the potential for bias induced by copy number amplification, we excluded 4 CpG sites on Chromosome 21, resulting in *m *= 96 CpG sites used for analysis. A clustering heatmap displaying the DNA methylation data appears in the Additional file 1. In one analysis, we compared cases and controls using the total leukocyte samples only, and in another we compared total leukocytes to T cells, pooling cases and controls. The Additional file 1. presents coefficient estimates.

#### Obesity in African Americans

Finally, we applied our method to an obesity data set [24] consisting of 7 lean African-Americans and 7 Obese African-Americans (GEO Accession number GSE25301). A clustering heatmap displaying the DNA methylation data appears in the Additional file 1. In this analysis, **z**consisted of obesity status.

#### Additional analyses

If the subject population for which **z **=** 0 **is sufficiently homogeneous with respect to blood cell distribution to admit sensible characterization of that distribution, then it is possible to recover estimates from $\hat{\Gamma }$. The Additional file 1 reports the results of such an analysis applied to the HNSCC case/control data set. Finally, we conducted an additional analysis where we took *S*_0_ to consist of only samples with pure CD4+ or CD8+ cells and *S*_1_ to consist only of samples having the less purified T-lymphocytes. For such *S*_1_, there were no covariates, so **z**consisted only of an intercept.

### Simulations

We conducted extensive simulation studies in order to verify the finite-sample statistical properties of our proposed methodology. Simulation parameters were obtained from the HNSCC data set, and most simulations assumed no sources of biological bias (DNA methylation changes arising from processes not mediated by the profiled leukocytes, including shifts in distribution within cell types not profiled). In every simulation, we specified *S*_0_ to consist of 5 B-cell samples, 10 granulocyte samples, 5 monocyte samples, 15 NK samples, 5 general “Pan-T” T-cell samples, 8 specific CD4+ T cell samples, and 2 specific CD8+ T cell samples. Estimates from the external validation set *S*_0_, described above, were used for mean methylation profiles among WBC types, using the *m *= 100 most informative CpG sites.

**Figure 3:**
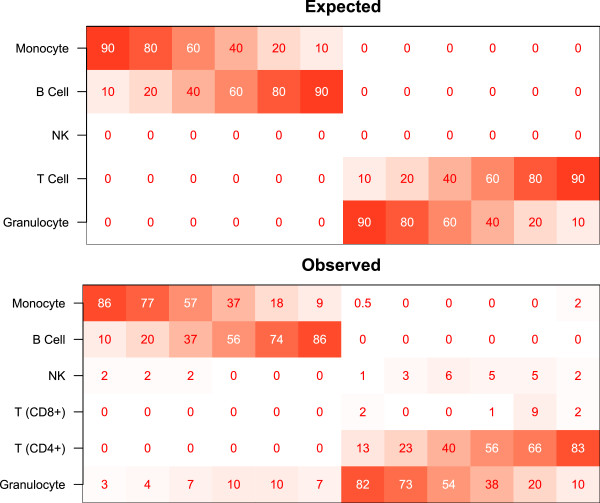
**Results of cell mixture reconstruction experiments validating prediction of individual profiles.** Expected and observed percentages of each cell type are shown by color (red=100, white=0) and text. Median root-mean-square-error over 12 samples had a median value of 8.2%, ranging from 5.4% to 11.6%.

We specified *n*_1_/2 cases and *n*_0_/2 controls, *n*_0_∈{100,200,500}. Among the controls, methylation profiles were generated by a white blood cell population of 7% B-cells, 62% granulocytes, 6% monocytes, 2% NK cells, and 13% were T-cells, of which 65% were CD4+ cells and 35% were CD8+ cells, and the remaining 5% were unspecified (and assumed to have mean methylation equal to that of the unsorted T-lymphocytes). Among cases, we specified one of the following scenarios: a 4% reduction in CD4+ cells, a 2% reduction in CD8+ cells, and an 8% increase in granulocytes (alternative with changes in both CD4+ and CD8+, “Strong Alternative I”); a 6% reduction in CD4+ cells, and an 8% increase in granulocytes (alternative with changes in CD4+ but not CD8+, “Strong Alternative II”); a weaker alternative with half the effects of Strong Alternative I (“Mixed Alternative” elaborated upon below); and two null scenarios with no changes in cell population, each with a different assumption about *δ*. Note that these changes reflect absolute changes in percentage points, not relative changes. Note also that these values were actually used to generate Dirichlet-distributed mixture weights for each simulated subject, with Dirichlet parameters equal to a precision parameter (100 corresponding to “precise” and 10 corresponding to“noisy”) times the mean weight described above. Residual effects $\xi_{i}^{\left(0\right)}$ for controls were set equal to 0.1 times estimated intercept estimate ${\hat{\mu }}_{1}$ obtained from the HNSCC data set, while residual effects $\xi_{i}^{\left(1\right)}$ for cases were set equal to 0.08 or 0.09 times ${\hat{\mu }}_{1}$ plus multiples 10*θ* of the column of $\hat{U}$ corresponding to case. The constants of proportionality 0.1, 0.08, and 0.09 were chosen to correspond to assumed contributions of *ξ* to an overall methylation signature presumed to be dominated by profiled populations of white blood cells in specified proportions, with 0.08 used for the strong alternatives and 0.09 used for the Mixed Alternative. The constant 10 was used to amplify the scale of *δ* so that its effect could be detected in simulation; note that $\hat{U}$ was orthogonal to the white blood cell profiles, by construction. The multiplier *θ *= 0 was used for strong alternatives, and the “Strong Null” case (i.e. no methylation differences between cases and controls) while *θ *= 0.5 was used for the Mixed Alternative, and *θ *= 1 was used for the “Mixed Null” with case/control differences not mediated by cellular population differences. A simple normal error structure for **e**_0*h *_and **e**_0*i*_ was specified, with no chip effects, but with variance equal to the sum of chip and residual variance estimated (individually for each CpG) for the HNSCC data. For each simulation, 50 bootstraps were used to estimate standard errors. 1000 simulations were run for each scenario.

## Results

In this section we report the results of the data analyses described above in Implementation, as well as the results of our simulation experiments.

### Cell mixture experiment

As Figure 3 suggests, accuracy is within 10%, and often less than 5%, with the largest errors occuring for granulocytes, as shown in Table 2. Note that the sum of the individual observed predictions for each individual profile ranged from 98.9% to 102.7% (data not shown), even though the constraints of the projection do not explicitly constrain the sum to 100%; this provides additional evidence that the DNA methylation profile captures a great deal of information about cell mixtures.

**Table 2:** Summary statistics for errors in cell mixture reconstruction results^*^

	**B cell**	**Granulocyte**	**Monocyte**	**NK**	**T cell**
minimum	0.0	0.3	0.0	0.0	0.0
median	0.1	6.5	1.1	2.1	0.3
maximum	5.5	10.0	4.1	6.4	5.3

^*^|Observed*%*−Expected*%*|.

### Head and neck cancer

Table 3 presents coefficient estimates $\hat{\Gamma }$ for case status, double-bootstrap bias estimates (estimates of bias arising from measurement error), as well as naive, single-bootstrap, and double-bootstrap standard error estimates. Each of these quantities is measured in percentage points (%). Estimates of bias arising from measurement error (i.e. substituting estimated quantities for known ones in a two-stage statistical procedure) were almost always less than half a percentage point, and for significant coefficient estimates, always towards the null. The proportion of CD4+ T-lymphocytes decreased in cases compared with controls, with a bias-corrected estimate of −10.4 percentage points and approximate 95% confidence interval (−13.1*%*,−3.3*%*); the proportion of NK cells decreased, with a bias-corrected estimate of -1.5 percentage points and 95% confidence interval (−2.2*%*,−0.75*%*); and the proportion of granulocytes increased, with a bias-corrected estimate of 7.6 percentage points and 95% confidence interval (4.2*%*,10.9*%*). There was also somewhat weaker evidence of an increase in CD8+ T-lymphocytes, with an estimate of 4.5 percentage points and 95% confidence interval (2.0*%*,7.0*%*). As reported in the complete set of results appearing in the Additional file 1, the proportion of CD4+ T-lymphocytes decreased by 3.3 percentage points (−4.4*%*,−2.2*%*) per decade of age, while CD8+ T-lymphocytes increased by 2.0 percentage point (1.0*%*,3.0*%*) per decade. All other coefficients were insignificant.

For this analysis, $R_{1,0}^{2}$ was estimated at 14.2%, while $R_{1,1}^{2}$ was estimated at 93.9%. Thus, a small but non-negligible proportion of total variation (systematic variation + unexplained biological heterogeneity + technical noise) appeared to be driven by changes in cell population between cases and controls and as a result of aging. Note that *SS*_*e*_ comprised 85% of total variation, so a substantial portion of variability in DNA methylation appeared to remain unexplained (presumably due, in large part, to technical noise). However, almost all of the systematic variation appeared to be explained by changes in cell population.

These results were consistent with previous studies, as HNSCC patients are known to display an absolute and relative increase in myeloid derived granulocytes [25] while also displaying an alteration in lymphoid T-cell homeostasis that leads to decreases in CD4+ T-cells [26,27]. In addition, the proportion of Treg cells (a subclass of CD4+ T cells) is known to decrease from infancy to adulthood [28].

The bias estimates obtained from the double-bootstrap procedure allow the correction of bias arising from measurement error. However, there is no statistical procedure for correcting the other possible sources of bias, those arising from changes in distribution among unprofiled cell types as well as non-immune-mediated methylation differences. The Additional file 1 presents a detailed sensitivity analysis, from which we show that the magnitude of the resulting bias is likely to be small, less than a percentage point.

### Ovarian cancer

Table 4 presents results for case-control status, with the remaining results appearing in the Additional file 1. $R_{1,0}^{2}$ was estimated at 17.8%, while $R_{1,1}^{2}$ was estimated at 86.1%.

**Table 3:** Estimates for HNSCC analysis (case vs. control)

	**Est**	**Bias**_**2**_	**SE**_**0**_	**SE**_**1**_	**SE**_**2**_	**P-value**
(Intercept, *γ*_0_)	−0.62	−0.02	0.41	0.52	0.52	0.23
B Cell	−0.45	0.04	0.30	0.77	0.76	0.55
Granulocyte	7.51	−0.07	0.50	1.73	1.71	<0.0001
Monocyte	0.49	0.10	0.50	0.47	0.48	0.31
NK	−1.43	0.06	0.56	0.37	0.38	0.00017
T Cell (cd4+)	−9.08	1.32	1.95	1.15	1.39	<0.0001
T Cell (cd8+)	3.06	−1.46	1.96	0.98	1.27	0.016

Est = Regression coefficient estimate (× 100%).

Bias_2_ = Double-bootstrap bias estimate (× 100%).

SE_0_ = Naive standard error (× 100%).

SE_1_ = Single-bootstrap standard error (× 100%).

SE_2_ = Double-bootstrap standard error (× 100%).

P-values were computed using SE_2_.

**Table 4:** Estimates for ovarian cancer analysis (case vs. control)

	**Est**	**Bias**_**2**_	**SE**_**0**_	**SE**_**1**_	**SE**_**2**_	**P-value**
(Intercept, *γ*_0_)	−0.05	−0.05	0.41	0.19	0.20	0.81
B Cell	−1.36	0.02	0.29	0.22	0.23	<0.0001
Granulocyte	8.97	−0.04	0.49	1.02	1.00	<0.0001
Monocyte	0.55	0.06	0.49	0.29	0.30	0.066
NK	−2.09	0.01	0.55	0.31	0.34	<0.0001
T Cell (cd4+)	−5.64	0.18	1.93	1.06	1.34	<0.0001
T Cell (cd8+)	−0.35	−0.17	1.93	0.95	1.19	0.77

Est = Regression coefficient estimate (× 100%).

Bias_2_ = Double-bootstrap bias estimate (× 100%).

SE_0_ = Naive standard error (× 100%).

SE_1_ = Single-bootstrap standard error (× 100%).

SE_2_ = Double-bootstrap standard error (× 100%).

P-values were computed using SE_2_.

Compared with controls, cases showed significant increases in granulocytes and significant decreases in B cells, NK cells, and CD4+ T cells. Cases also showed marginally significant increases in monocytes. These results are consistent with previous literature, where it has been demonstrated that ovarian cancer patients experience decreases in B and T lymphocytes [29-31], increases in monocytes [29,30] and (somewhat equivocally) increases in eosinophil granulocytes [30]. Additionally, there were significant systematic decreases in CD4+ T cells with increasing age, with a gradient consistent in direction and somewhat consistent in magnitude with the corresponding effect found in the HNSCC data set. Though most of the CD8+ T cell coefficients for age were not significant, they were all positive, with gradient consistent in direction and somewhat consistent in magnitude with the corresponding effect found in the HNSCC data set. As reported in the Additional file 1, no bisulfite conversion coefficient was significant, and all coefficients were of small magnitude (generally less than 1 percentage point per standard deviation).

### Down syndrome

The only significant difference between cases and controls was in B cell distribution, with bias-corrected estimated decrease of 4.8%, 95% confidence interval (−6.2%, −3.5%). This result is consistent with known immune characteristics of Down Syndrome, including deficiencies in both B and T cells [32,33]. However, in the comparison between total leukocytes and T cells, all coefficients except B Cell and NK were highly significant, in directions consistent with comparison of a sample of purified T cells to a generic whole blood sample. In fact, an estimate of the cellular composition of the T cell samples can be obtained by a simple linear transformation of *Γ *estimates (adding intercept terms with the T cell coefficients); this operation produces values that are not significantly distinct from zero for all cell types except CD4+ and CD8+, whose bias-corrected estimates were, respectively, 75.9%, 95% confidence interval (67%,85%) and 8.6%, 95% confidence interval (0%,17%), consistent with the known distribution of these T cells. For the analysis of case vs. control within total leukocytes, $R_{1,0}^{2}$ was estimated at 4.5%, while $R_{1,1}^{2}$ was estimated at 67.6%. For the analysis of total leukocyte vs. T cell with pooled cases and controls, $R_{1,0}^{2}$ was estimated at 81.4%, while $R_{1,1}^{2}$ was estimated at 98.9%. The latter set of coefficients of determination indicate that a substantial portion of variation is explained by composition of leukocytes, which is the expected result for such an analysis.

### Obesity in African Americans

Obese subjects had an estimated increase of 12 percentage points in granulocytes, bias-corrected 95% confidence interval (3.4%, 20%) and an estimated decrease of 4 percentage points in NK cells, bias-corrected 95% confidence interval (-7.7%,-0.9%). No significant differences were found for other blood cell types. Note that the specific immunological differences estimated by the method are consistent with known immunological perturbations associated with type II diabetes [9,10]. Complete results are provided in the Additional file 1.

### Additional analyses

We obtained the following unnormalized bias-corrected estimates: 69.0% CD4+, 95% CI (54%,84%), and 32.5% CD8+, 95%CI (19%,46%). This is consistent with known proportions of these specific cell types among T lymphocytes.

### Results of simulations

Table 5 presents results for *n*_1_ = 200 with precise mixture weights (small within-status heterogeneity in distribution), while Table 6 presents results for *n*_1_ = 200 with noisy mixture weights (larger within-status heterogeneity). The tables show mean estimate, simulation standard deviation, median estimates for the three types of proposed standard errors, and proportion of p-values (obtained from z-scores constructed using the double-bootstrap standard error) falling below *α *= 0.05 and *α *= 0.01. In all cases, the bias in estimation was negligible. Both bootstrap procedures produced similar standard error estimates, which were close to the simulation standard deviation but often quite different from the naive standard error estimate. Under null scenarios, the rejection probabilities were tolerably close to their nominal values, and for alternatives, power could be quite high, even with this modest design. Results for the coefficients of determination are provided in the Additional file 1. Scenarios with *n*_1_∈{100,500} produced similar results, with simulation standard deviations and power adjusted accordingly, but still having practical utility.

**Table 5:** Simulation Results (Precise Mixtures, *n*_1_ = 200)

**Strong Alternative I (*θ = *0)**								
	**Truth**	**Est**	**SD**	**SE**_**0**_	**SE**_**1**_	**SE**_**2**_	**pow(0.05)**	**pow(0.01)**
B Cell	0.0	0.07	1.00	0.92	0.97	0.98	0.057	0.018
Granulocyte	8.0	8.02	0.73	0.39	0.73	0.73	1.000	1.000
Monocyte	0.0	0.01	0.48	0.43	0.47	0.47	0.055	0.013
NK	0.0	−0.09	1.08	1.02	1.02	1.05	0.066	0.015
T Cell (cd4+)	−4.0	−4.06	0.81	0.80	0.78	0.81	0.999	0.989
T Cell (cd8+)	−2.0	−1.93	0.83	0.81	0.78	0.81	0.653	0.419
**Strong Alternative II (*θ = *0)**								
	**Truth**	**Est**	**SD**	**SE**_**0**_	**SE**_**1**_	**SE**_**2**_	**pow(0.05)**	**pow(0.01)**
B Cell	0.0	0.00	0.97	0.92	0.97	0.99	0.048	0.016
Granulocyte	8.0	8.00	0.71	0.39	0.72	0.72	1.000	1.000
Monocyte	0.0	0.03	0.48	0.42	0.47	0.47	0.063	0.016
NK	0.0	0.03	1.04	1.02	1.01	1.05	0.052	0.014
T Cell (cd4+)	−6.0	−5.83	0.76	0.80	0.77	0.80	1.000	1.000
T Cell (cd8+)	0.0	−0.22	0.81	0.81	0.80	0.81	0.064	0.014
**Mixed Alternative (*θ = *0.5)**								
	**Truth**	**Est**	**SD**	**SE**_**0**_	**SE**_**1**_	**SE**_**2**_	**pow(0.05)**	**pow(0.01)**
B Cell	0.0	−0.02	1.02	1.10	0.96	0.98	0.065	0.011
Granulocyte	4.0	3.99	0.75	0.47	0.73	0.73	1.000	0.995
Monocyte	0.0	0.02	0.49	0.51	0.47	0.47	0.060	0.015
NK	0.0	0.04	1.05	1.22	1.01	1.04	0.054	0.009
T Cell (cd4+)	−2.0	−2.07	0.82	0.96	0.79	0.83	0.695	0.471
T Cell (cd8+)	−1.0	−0.95	0.82	0.96	0.78	0.82	0.203	0.082
**Mixed Null (*θ = *1)**								
	**Truth**	**Est**	**SD**	**SE**_**0**_	**SE**_**1**_	**SE**_**2**_	**pow(0.05)**	**pow(0.01)**
B Cell	0.0	0.00	1.04	1.58	0.96	1.02	0.066	0.017
Granulocyte	0.0	0.03	0.73	0.67	0.74	0.74	0.055	0.014
Monocyte	0.0	−0.01	0.47	0.73	0.47	0.48	0.054	0.013
NK	0.0	−0.01	1.12	1.76	1.01	1.09	0.063	0.014
T Cell (cd4+)	0.0	0.01	0.87	1.38	0.80	0.90	0.054	0.013
T Cell (cd8+)	0.0	−0.02	0.88	1.39	0.79	0.89	0.057	0.015
**Strong Null (*θ = *0)**								
	**Truth**	**Est**	**SD**	**SE**_**0**_	**SE**_**1**_	**SE**_**2**_	**pow(0.05)**	**pow(0.01)**
B Cell	0.0	−0.01	0.99	0.90	0.96	0.96	0.068	0.014
Granulocyte	0.0	0.03	0.72	0.38	0.74	0.73	0.052	0.013
Monocyte	0.0	−0.01	0.47	0.42	0.47	0.47	0.055	0.013
NK	0.0	−0.01	1.06	1.00	1.01	1.02	0.059	0.020
T Cell (cd4+)	0.0	0.00	0.81	0.78	0.80	0.82	0.054	0.013
T Cell (cd8+)	0.0	−0.01	0.81	0.79	0.79	0.80	0.054	0.015

Est = Men regression coefficient estimate (× 100%); SD = SD regression coefficient estimate (× 100%).

SE_0_ = Naive standard error (× 100%); SE_1_ = Single-bootstrap standard error (× 100%).

SE_2_ = Double-bootstrap standard error (× 100%).

pow(*α*) =* Pr*{_*P*2_ <* α*}, where *P*_2_ is the p-value computed from SE_2_.

**Table 6:** Simulation Results (Noisy Mixtures, *n*_1_ = 200)

**Strong Alternative I (*θ = *0)**								
	**Truth**	**Est**	**SD**	**SE**_**0**_	**SE**_**1**_	**SE**_**2**_	**pow(0.05)**	**pow(0.01)**
B Cell	0.0	−0.06	1.39	0.92	1.36	1.34	0.065	0.019
Granulocyte	8.0	7.87	2.02	0.39	2.00	1.99	0.974	0.897
Monocyte	0.0	0.05	1.03	0.42	1.04	1.02	0.049	0.012
NK	0.0	−0.02	1.21	1.02	1.16	1.18	0.061	0.010
T Cell (cd4+)	−4.0	−4.00	1.23	0.79	1.21	1.22	0.903	0.739
T Cell (cd8+)	−2.0	−1.97	1.05	0.80	1.02	0.98	0.517	0.298
**Strong Alternative II (*θ = *0)**								
	**Truth**	**Est**	**SD**	**SE**_**0**_	**SE**_**1**_	**SE**_**2**_	pow(0.05)	pow(0.01)
B Cell	0.0	−0.08	1.38	0.92	1.36	1.34	0.063	0.017
Granulocyte	8.0	7.90	2.03	0.39	1.99	1.98	0.973	0.905
Monocyte	0.0	0.10	1.07	0.42	1.04	1.02	0.054	0.019
NK	0.0	0.02	1.17	1.02	1.14	1.18	0.053	0.009
T Cell (cd4+)	−6.0	−5.70	1.19	0.80	1.13	1.16	0.999	0.986
T Cell (cd8+)	0.0	−0.23	1.08	0.81	1.10	1.04	0.066	0.015
**Mixed Alternative (*θ = *0.5)**								
	**Truth**	**Est**	**SD**	**SE**_**0**_	**SE**_**1**_	**SE**_**2**_	**pow(0.05)**	**pow(0.01)**
B Cell	0.0	0.05	1.42	1.10	1.34	1.34	0.066	0.016
Granulocyte	4.0	4.00	2.01	0.47	2.02	2.01	0.500	0.291
Monocyte	0.0	0.01	1.06	0.51	1.03	1.02	0.072	0.020
NK	0.0	−0.02	1.24	1.22	1.13	1.16	0.064	0.013
T Cell (cd4+)	−2.0	−2.11	1.30	0.95	1.26	1.28	0.391	0.191
T Cell (cd8+)	−1.0	−0.94	1.08	0.96	1.05	1.02	0.163	0.052
**Mixed Null (*θ = *1)**								
	**Truth**	**Est**	**SD**	**SE**_**0**_	**SE**_**1**_	**SE**_**2**_	**pow(0.05)**	**pow(0.01)**
B Cell	0.0	0.06	1.41	1.59	1.36	1.37	0.062	0.016
Granulocyte	0.0	0.04	2.08	0.67	2.06	2.05	0.056	0.008
Monocyte	0.0	−0.02	1.05	0.73	1.03	1.03	0.058	0.020
NK	0.0	0.01	1.26	1.76	1.14	1.22	0.066	0.011
T Cell (cd4+)	0.0	−0.01	1.42	1.38	1.31	1.36	0.067	0.016
T Cell (cd8+)	0.0	0.00	1.19	1.39	1.08	1.10	0.073	0.011
**Strong Null (*θ = *0)**								
	**Truth**	**Est**	**SD**	**SE**_**0**_	**SE**_**1**_	**SE**_**2**_	**pow(0.05)**	**pow(0.01)**
B Cell	0.0	0.06	1.37	0.91	1.36	1.32	0.065	0.017
Granulocyte	0.0	0.03	2.07	0.38	2.06	2.05	0.055	0.009
Monocyte	0.0	−0.02	1.04	0.42	1.03	1.02	0.057	0.021
NK	0.0	0.01	1.19	1.01	1.14	1.16	0.053	0.018
T Cell (cd4+)	0.0	−0.04	1.38	0.79	1.31	1.31	0.069	0.015
T Cell (cd8+)	0.0	0.01	1.11	0.79	1.08	1.03	0.065	0.016

Est = Mean regression coefficient estimate (× 100%); SD = SD regression coefficient estimate (× 100%).

SE_0_ = Naive standard error (× 100%); SE_1_ = Single-bootstrap standard error (× 100%).

SE_2_ = Double-bootstrap standard error (× 100%).

pow(*α*) =* Pr*{_*P*2_ <* α*}, where *P*_2_ is the p-value computed from SE_2_.

## Discussion

In this paper, we employ the concept of DMRs as markers of immune cell identity using a high density methylation platform, and propose a set of analytical tools for estimating the proportions of immune cells in unfractionated whole blood. The backbone of the approach is the DNA methylation signature of each of the principal immune components of whole blood (B cells, granulocytes, monocytes, NK cells, and T cells subsets). The examples we have provided above serve to illustrate that our proposed methodology produces parameter estimates consistent with the literature, thus validating its utility.

Our proposed method resembles regression calibration, where we consider a methylation signature to be a high-dimensional multivariate surrogate for the distribution of white blood cells. In turn, this distribution is of interest for predicting or modeling disease states. As a surrogate, the DNA methylation signature is assumed to be a highly correlated, yet imperfect, measure of leukocyte distribution, and thus fits into the framework of measurement error models, where the use of a noisy surrogate marker to investigate an association with a disease outcome of interest results in biased estimates, unless internal or external validation data can be obtained to “calibrate” the model and correct the bias [12]. However, in this case, the problem is complicated by the extremely high dimension of the surrogate. Measurement error problems are typically formulated as a set of relationships between **z**, the disease outcome (e.g. case/control status), *ω*, the gold standard (e.g. leukocyte distribution), and **Y**, the surrogate (e.g. DNA methylation). Of interest is E(**z **|* ω*), which may be difficult to estimate due to the cost or logistical complications involved in obtaining *ω *in a large number of samples. Typically, it is possible to collect sufficient data for modeling E(**z **|** Y**), which provides information about E(**z **|* ω*) through the (often imperfect) association E(**Y **|* ω*), which is inferred from an external validation sample [12,34]. Unfortunately, the high-dimensional nature of **Y** renders E(**z **|** Y**) difficult to formulate. While multivariate methods of measurement error correction exist, even in a high-dimensional context [35], they require an explicit specification of E(**z**|**Y**), requiring a large number of parameters even for a main effects regression model, and many more in order to account for interactions. This becomes unwieldy when each component of **Y** contributes a small amount of information about **z**, and both dimension-reduction strategies and constrained regression strategies entail substantial loss of information and may be extremely computationally intensive. Existing measurement error formulations [34,35] would have required us to specify a logistic regression model for case/control status, conditional on DNA methylation signature, a computationally difficult task that would have extreme vulnerability to model mis-specification. On the other hand, our method requires specification of E(**Y **|** z**), which is natural and straightforward. Note that in some treatments of regression calibration, E(*ω *|** Y**) is used as a surrogate for *ω* in regression models for **z**[12]; our treatment essentially assumes a linear form for E(**Y **|* ω*) and effectively obtains E(*ω *|** Y**) by projecting **Y **onto the column space of resulting matrix. We note that it is possible using existing methods to qualitatively describe immune response contributions to DNA methylation. This is typically done by conducting a pathway analysis along the lines of one of the methods described in [36], the best option of which is Gene Set Enrichment Analysis (GSEA) [37]. For example, Teschendorff et al. (2009) [22] use GSEA to qualitatively motivate an immunological explanation. However, these methods do not directly quantify the immunological contribution.

An important consideration in the measurement error literature is that of transportability of model parameters [38]. In our setting, an important consideration is whether the methylation profiles obtained from the purified blood cells used to assemble *S*_0_ would be representative of the white blood cells measured within *S*_1_. Because of the biological assumptions inherent in the DMR literature and underlying current understanding of hematopoeisis and lineage commitment, this assumption is reasonable, provided our method is used to detect abnormal mixtures of normal white blood cells. However, methylation abnormalities in the white blood cells themselves constitute a form of non-cell mediated alteration (in the sense of the term we have been using), and contribute to bias in our methods, as described briefly above and in detail in the Additional file 1.

Note that our formulation respects the study design (DNA methylation assay data collected after sampling from phenotype groups). An alternative strategy outside the measurement error literature but within the larger missing-data literature might have been the use of an Expectation-Maximization (EM) algorithm to integrate over the missing data *ω*[39]. However, by design, the distribution of *ω *varied substantially between the data sets *S*_0_ and *S*_1_, severely complicating the approach; notably, an would be the introduction of feedback from *S*_1_ to *S*_0_, contaminating the gold-standard status of *S*_0_. An alternative, might be the use of an empirical Bayes procedure, reminiscent of existing mixture-model approaches [40]. However, difficulty in specifying the distribution of “remainder terms” (denoted as *ξ*above) render this approach untenable, and in simulations (not presented), attempts to impute *ω*among *S*_1_ samples using parameters obtained from *S*_0_ samples resulted in extremely biased estimates of *ω*.

The most significant aspect of the current study is our development of a method for inferring changes in the distribution of white blood cell types between different human populations (e.g. cases and controls) using DNA methylation signatures; an approach guided by an external validation set consisting of methylation profiles from purified white blood cell components. DNA methylation in peripheral blood is a potentially powerful new biomarker for clinical and epidemiological investigation. By example, numerous studies have now attempted to distinguish cancer cases from controls using whole peripheral blood assayed via DNA methylation arrays, including ovarian [22], bladder [41], and pancreatic [42] cancers. While these studies have demonstrated good to excellent discrimination of cases from controls, sound evidence for a biological mechanism has been elusive. Presumably, disease associated alterations in blood methylation have several etiological components driven by inherent genetic, environmental and disease specific factors. Given the known developmental associated differences in DNA methylation among specific blood cell types, changes in the distributions of blood cell types alone could account for disease associated DNA methylation. While numerous authors provide a qualitative discussion that includes the possibility of immune-related DNA methylation differences (e.g. [22]), none to date has specifically quantified the contribution from immune response. On the other hand, the many diverse types of immune cells in blood make this issue highly complex and problematic to tackle using single cell type assays. Therefore, it is crucial to the development of this new avenue of biomarker research to delineate effects due to the immune cell distribution itself from other “non cell type” alterations in DNA methylation. We term the differences among human populations attributed to cell distributions to be “immunologically mediated”. Our solution to partition this component of variation in methylation from other determinants are multivariate analytic tools including regression coefficients and associated inference, as well as coefficients of determination measures. Taken together these provide a means for evaluating whether the observed DNA methylation differences are due to an immunologically mediated response.

In our Additional file 1 we provide a detailed analysis of potential sources of bias in our analysis. One obvious biological source of bias is age of the subjects contributing cells for validation. At certain CpG loci, DNA methylation is known to change with age [43], especially in T cells [44]. In the Additional file 1 we demonstrate that any age-related associations with DNA methylation in our top 100 CpGs were too weak to be detected with the current validation sample, and thus unlikely to bias the results of our analyses (notably age coefficients provided for the HNSCC example). However, we remark that with larger sample sizes, adjustments for age can be incorporated with an appropriate additional term in the linear model (1) for **Y**_0*h*_.

Similar methods based on mRNA have been employed [13-15]. The statistical principles described in this article would apply, wholesale, to mRNA expression profiles, but with two cautionary statements. The first is mathematical: mRNA is typically analyzed on a logarithmic scale, yet the assumptions of the proposed methodology involve linearity on an arithmetic scale, since the mixing coefficients are assumed to act linearly on absolute numbers of nucleic acid molecules; thus, the proposed methods would require analysis of untransformed fluorescence intensities, whose skewed distributions would result in numerical instabilities. The second is biological: there is no necessarily linear relationship between cell number and mRNA copies, since proteins may be translated as a consequence of an initial burst of mRNA transcription upon cellular development, after which significant mRNA degradation is possible. In contrast, one would expect the average beta value provided by Illumina bead-array products (and similar quantities) to scale in proportion to the actual fraction of methylated nucleic acids; in addition, an assumption of two DNA molecules per cell seems biologically reasonable. In the Additional file 1 we provide an example of an application of our methods using mRNA data.

Going forward there are two issues that require further experimental and analytical refinement. First, although the current studies suggest group level comparisons of blood cell DNA methylation can reveal important immune alterations, it will be important to provide methods for individual level immune cell profiling, since clinical and detailed analytical epidemiologic applications that examine individual risk factor information will be the subject of future studies. As we have demonstrated above, individual immune profiles are theoretically achievable but will require extensive validation, with a wide array of mixture combinations, before gaining widespread acceptance. Secondly, there is intense interest in minor immune cell fractions and their role in disease, though the signal strength of cell types comprising < 5% of the total white cell compartment may be difficult to quantitate. Examples of such cell types include the regulatory T-cell or NK cell fractions, which are implicated in autoimmune and malignant diseases. Optimization of platforms for technical sensitivity to minor subtypes combined with statistical optimization of signature recognition are needed to enhance the approach for testing highly targeted immune hypotheses.

## Conclusions

The method we present here has potentially far reaching implications for rapid, simple and complete assessment of the composition of human white blood cell populations, i.e. the immune profile. Currently, assessment of the cellular composition of peripheral blood cannot be accomplished without the use of freshly drawn venous blood that is immediately prepared in a specially equipped laboratory. A complete assessment of the entire immune profile requires extensive flow cytometric measurements based on protein epitopes on leukocyte membranes that distinguishes subtypes of immune cells that are either too rare or too similar in appearance to be distinguished using simple microscopic approaches. In particular, flow cytometry is limited by the following: (i) cells must be separated, requiring large volumes of fresh cells; (ii) detection can be accomplished only by the fluorescent antibody tags available, which require expensive technology to read; (iii) the outer cell membrane must be intact, mandating limited utility in many instances (particularly in research). In contrast, our method requires the application of these labor-intensive or expensive steps only in the construction of the validation set *S*_0_, which need only be developed once. Once *S*_0_ is available, subsequent interrogation is based on the chemically stable CpG methylation of DNA; thus our method obviates the need for fresh blood and the preservation of labile protein epitopes. It is also able to simultaneously assess all of the individual components of the peripheral blood using a highly multiplexed molecular platform and is thus very straightforward logistically. Furthermore, the statistical methodology presented here can be implemented easily with the instrumental output of the methylation arrays, which simplifies the interpretation of the immune profile data from the operators point of view. This method can be immediately deployed in a research framework to cost effectively assess human immune profiles (in fresh or archival samples), exploring their potential as biomarkers, and addressing key questions regarding disease pathogenesis. Furthermore, our approach is readily suited for rapid translation to a broad base of clinical applications such as disease monitoring, diagnosis, prognosis, and response to therapy.

Our approach makes research on biobanked specimens possible, now making a vast array of prospective studies that could not otherwise be done, possible. Software and sample data are provided in Additional file 2.

## Abbreviations

CTL: Cytotoxic T-cells; CpG: Cytosine-phosphate-guanine; DMR, Differentially methylated region; HNSCC: Head and neck squamous cell carcinoma; NK: Matural killer.

## Competing interests

A patent is pending on the work contained in this article. The authors have no other competing interests.

## Authors’ contributions

EAH conceived of the statistical model, developed the algorithms, conducted the simulations, applied the methods to proprietary and publicly available data sets and authored major parts of the manuscript. WPA conducted the laboratory experiments and authored parts of the manuscript. JKW and KTK conceived of the laboratory experiments and provided grant support for the research. DCK provided indispensible feedback on statistical methodology. BCC, CJM, and HHN provided indispensible feedback on scientific issues and interpretation. All authors read and approved the final manuscript.

## Supplementary Material

Additional file 1 **Houseman-WBC-BMCBioinformatics- Supplement.pdf.** Additional theoretical details, simulation descriptions and results, and additional figures and result tables [43-52]. Click here for file

Additional file 2 **Houseman-WBC-BMCBioinformatics-Software-v2. **Sample R software (compressed). Click here for file
