# Supplementary material for: DNA methylation arrays as surrogate measures of cell mixture distribution
Source: BMC Bioinformatics. 2012 May 8;13:86. doi: 10.1186/1471-2105-13-86 (PMC3532182; doi:10.1186/1471-2105-13-86)
Supplement: Additional file 1 — Houseman-WBC-BMCBioinformatics- Supplement.pdf. Additional theoretical details, simulation descriptions and results, and additional figures and result tables [43-52]. [file 1471-2105-13-86-S1.pdf]

DNA Methylation Arrays as Surrogate Measures of Cell Mixture Distribution: Supplementary Material

E. Andrés Houseman[1,*], William P. Accomando[2], Devin C. Koestler[3], Brock C. Christensen[3], Carmen J. Marsit[3], Heather H. Nelson[4], John K. Wiencke[5], Karl T. Kelsey[2,6]
1 College of Public Health and Human Sciences, Oregon State University, Corvallis, Oregon, USA
2 Department of Pathobiology, Brown University, Providence, Rhode Island, U.S.A.
3 Section of Biostatistics and Epidemiology, Dartmouth Medical School, Hanover, New Hampshire, U.S.A.
4 Department of Epidemiology, University of Minnesota, Minneapolis, Minnesota, U.S.A.
5 Department of Neurological Surgery, University of California San Francisco, San Francisco, California, U.S.A.
6 Department of Epidemiology, Brown University, Providence, Rhode Island, U.S.A.
∗ E-mail: andres.houseman@oregonstate.edu

S1 Supplement to Methods

S1.1 General Designs for S_0

Because the cell types assembled in S_0 potentially involve hierarchical relationships corresponding to cell lineage, designs that are more general than a one-way ANOVA parameterization may be necessary for $\mathbf{w}$. If cell-type interpretations can be extracted from S_0 via some $d_0 \times d_0^*$ contrast matrix $\mathbf{L}$ (i.e. $\mathbf{B}_0\mathbf{L}$ identifies the mean methylation for d_0^* cell types), then interpretations can be obtained by simply replacing $\widehat{\mathbf{B}}_0$ with $\widehat{\mathbf{B}}_0\mathbf{L}$ in the projection used to estimate $\boldsymbol{\gamma}_0$ and $\boldsymbol{\Gamma}$ and their standard errors.

As an example, consider the case of CD4+ and CD8+ T cells, both of which are the primary components of the T-lymphocyte group. Suppose a sample of purified CD4+ T cells is prepared, another sample of purified CD8+ T cells, and finally a sample of T-lymphocyte cells that have not been purified to more specific lineages. Such was the case for S_0 in the examples. The CD4+ sample may be identified as $\mathbf{w}_{0h} = (1,1,0)^{\mathrm{T}}$, the CD8+ sample as $\mathbf{w}_{0h} = (1,0,1)^{\mathrm{T}}$, and the latter, less specific sample as $\mathbf{w}_{0h} = (1,0,0)^{\mathrm{T}}$. Then an appropriate contrast $\mathbf{L}$ for identifying CD4+ and CD8+ samples would be constructed as a 3×2 matrix with columns $(1,1,0)^{\mathrm{T}}$ and $(1,0,1)^{\mathrm{T}}$. This approach was used in all of the data examples, and was also employed in the simulations.

S1.2 Estimation Details

Here we provide details on estimation, introducing a two-stage estimation procedure. The first stage of analysis involves estimation of $\mathbf{B}_0$ and $\mathbf{B}_1$ by appropriate linear models, e.g. ordinary least squares (OLS) regression estimator $\widehat{\mathbf{B}}_0^{\mathrm{T}} = \left[\sum_{h=1}^{n_0} \mathbf{z}_{0h}\mathbf{z}_{0h}^{\mathrm{T}}\right]^{-1} \left[\sum_{h=1}^{n_0} \mathbf{z}_{0h}^{\mathrm{T}}\mathbf{Y}_{0h}^{\mathrm{T}}\right]$ and a similar estimator for $(\widehat{\boldsymbol{\mu}}_1, \widehat{\mathbf{B}}_1)^{\mathrm{T}}$; a procedure such as *limma*; or else locus-by-locus linear mixed effects models that adjust for technical (e.g. chip) effects. The second stage of analysis, estimation of $\widehat{\boldsymbol{\gamma}}_0$ and $\widehat{\boldsymbol{\Gamma}}$, proceeds as follows:

$$(\widehat{\boldsymbol{\gamma}}_0, \widehat{\boldsymbol{\Gamma}}^{\mathrm{T}})^{\mathrm{T}} = \widehat{\mathbf{B}}_1^{\mathrm{T}}\tilde{\mathbf{B}}_0(\tilde{\mathbf{B}}_0^{\mathrm{T}}\tilde{\mathbf{B}}_0)^{-1}, \tag{S1}$$

where $\tilde{\mathbf{B}}_0 = (\mathbf{1}_m, \widehat{\mathbf{B}}_0)$. Let $\widehat{\mathbf{r}}_\gamma = \widehat{\mathbf{B}}_1 - \mathbf{1}_m\widehat{\boldsymbol{\gamma}}_0 - \widehat{\mathbf{B}}_0\widehat{\boldsymbol{\Gamma}}$, $\widehat{\boldsymbol{\Sigma}}_\gamma \equiv (\widehat{\sigma}_{rs}^{(\gamma)})_{rs} = (m - d_0 - 1)^{-1}\widehat{\mathbf{r}}_\gamma^{\mathrm{T}}\widehat{\mathbf{r}}_\gamma$, $\mathbf{V}_0 = m(\tilde{\mathbf{B}}_0^{\mathrm{T}}\tilde{\mathbf{B}}_0)^{-1}$, and $\mathbf{V}_0 = (v_{rs}^{(0)})_{rs}$. Naive standard error estimates for the $(r,s)^{th}$ element of $(\widehat{\boldsymbol{\gamma}}_0, \widehat{\boldsymbol{\Gamma}}^{\mathrm{T}})$ can be obtained by computing $(m^{-1}v_{ss}^{(0)}\widehat{\sigma}_{rr}^{(\gamma)})^{1/2}$. However, the naive standard error estimates fail to account for the variability in estimating $\widehat{\mathbf{B}}_0$ and $\widehat{\mathbf{B}}_1$, and are consequently biased, as we demonstrate in the simulations.

A simple alternative is to use a nonparametric bootstrap procedure. For each bootstrap iteration t, we sample with replacement from S_1, (or sample errors in a manner consistent with a hierarchical experimental design, e.g. taking into account chip effects), to obtain $S_1^{(t)}$. From $S_1^{(t)}$, we obtain an estimate $\widehat{\mathbf{B}}_1^{(t)}$, after which we compute $\widehat{\boldsymbol{\gamma}}_0^{(t)}$ and $\widehat{\boldsymbol{\Gamma}}^{(t)}$ by replacing $\widehat{\mathbf{B}}_1$ with $\widehat{\mathbf{B}}_1^{(t)}$ in (S1). After resampling a large number T times, standard errors can easily be obtained empirically from the bootstrap sets $\{\widehat{\boldsymbol{\gamma}}_0^{(t)}\}_{t=1,...,T}$ and $\{\widehat{\boldsymbol{\Gamma}}^{(t)}\}_{t=1,...,T}$. We will call this method of estimation the "single bootstrap" to distinguish it from an alternative that accounts for variability in estimation of $\widehat{\mathbf{B}}_0$ as well.

Because S_0 will typically consist of small sample sizes per cell type, a nonparametric bootstrap procedure for estimating variation in $\widehat{\mathbf{B}}_0$ may not perform well. We therefore use a parametric bootstrap. Let $\boldsymbol{\Omega}_j$ be the variance-covariance matrix for the jth row of $\widehat{\mathbf{B}}_0$. We form a resampled matrix $\widehat{\mathbf{B}}_0^{(t)}$ by adding, to each row j of $\widehat{\mathbf{B}}_0$, a zero-mean multivariate normal vector with variance-covariance $\boldsymbol{\Omega}_j$, or a corresponding multivariate t-distribution with $n_0 - d_0$ degrees of freedom. Then we compute $\widehat{\boldsymbol{\gamma}}_0^{(t)}$ and $\widehat{\boldsymbol{\Gamma}}^{(t)}$ from (S1) by replacing $\widehat{\mathbf{B}}_0$ with $\widehat{\mathbf{B}}_0^{(t)}$ (in addition to the previously mentioned replacement). We will refer to this method as the "double bootstrap". Note that the double bootstrap ignores correlation between CpG sites within a single validation sample, but given the relative purity assumed for these samples and adequate correction for technical effects, this should be reasonable to first order. As we demonstrated in the data examples and simulations, there is negligible difference between the single and double bootstrap, so the incorporation of additional complexity to model cross-CpG correlations is unlikely to produce much benefit. However, the double-bootstrap has the additional benefit over the single-bootstrap, in that it can be used to assess bias due to measurement error (variability) in $\widehat{\mathbf{B}}_0$.

S2 Bias

Here we describe several potential sources of bias in the proposed methodology, presenting the underlying theoretical considerations, as well as a sensitivity analysis based on these considerations. The first form of bias, from measurement error, is easily assessed using the double-bootstrap procedure described above. Below we provide a detailed mathematical treatment of possible biases induced by biological non-orthogonality. We end with a discussion of potential bias resulting from age-related changes in DNA methylation measured in the validation specimens.

S2.1 Theory

Consider a univariate z_{1i} representing case/control status, where $\boldsymbol{\delta} \equiv \boldsymbol{\xi}^{(1)} - \boldsymbol{\xi}^{(0)} = \mathbf{B}_0\boldsymbol{\alpha}$ for some $d_0 \times 1$ vector $\boldsymbol{\alpha} \neq \mathbf{0}$; i.e. $\boldsymbol{\delta}$ is the mean difference in DNA methylation between a case and control, contributed by cell mixtures that remain uncharacterized or non-cell-specific methylation. In such a situation, there will be a bias equal to $\boldsymbol{\alpha}$ in estimating the mixture differences. Non-orthogonal $\boldsymbol{\delta}$ may arise from two distinct sources. One occurs when some cell types have not been profiled in S_0, so that $\sum_{l=0}^{d_0} \omega_l^{(z)} < 1$. The other may arise when some non-cell-mediated biological process (i.e. distinct from a change in cellular mixtures) nevertheless results in methylation profiles that appear similar to those that distinguish cell types profiled in S_0. To this end, we elaborate model (4) in the main text as follows:

$$\mathrm{E}(\mathbf{Y}_{1i}|\mathbf{z}_{1i1} = z) = \sum_{l=1}^{d_0}(\mathbf{B}_0\boldsymbol{\varepsilon}_l + \boldsymbol{\lambda}_l^{(z)})\omega_l^{(z)} + \sum_{q=1}^{Q}(\tilde{\boldsymbol{\mu}}_q + \tilde{\boldsymbol{\lambda}}_q^{(z)})\tilde{\omega}_q^{(z)}, \tag{S2}$$

where $q \in \{1, ..., Q\}$ indexes unprofiled cell types (or free DNA), each with methylation profile $\tilde{\boldsymbol{\mu}}_q$, and in mixture proportions $\omega_l^{(z)}$ and $\tilde{\omega}_q^{(z)}$, $\sum_{l=1}^{d_0} \omega_l^{(z)} + \sum_{q=1}^{Q} \tilde{\omega}_q^{(z)} = 1$. Here $\boldsymbol{\lambda}^{(z)}$ denotes an "abnormal", or at least non-functional, non-cell-mediated process that is specific to disease status (but may affect different

cell types in different degrees of intensity). Let $\mathbf{P} = (\tilde{\mathbf{B}}_0^{\mathrm{T}}\tilde{\mathbf{B}}_0)^{-1}\tilde{\mathbf{B}}_0^{\mathrm{T}}$, and denote difference between case and control parameters using Δ, e.g. $\Delta\omega_l = \omega_l^{(1)} - \omega_l^{(0)}$ and $\Delta\mathrm{E}(\mathbf{Y}_{1i}) = \mathrm{E}(\mathbf{Y}_{1i}|\mathbf{z}_{1i1} = 1) - \mathrm{E}(\mathbf{Y}_{1i}|\mathbf{z}_{1i1} = 0)$. It follows from (S2) that

$$\mathbf{P}\Delta\mathrm{E}(\mathbf{Y}_{1i}) = \sum_{l=1}^{d_0} \boldsymbol{\varepsilon}_l \Delta\omega_l + \sum_{q=1}^{Q} \mathbf{P}\tilde{\boldsymbol{\mu}}_q \Delta\tilde{\omega}_q + \sum_{l=1}^{d_0} \mathbf{P}\Delta(\boldsymbol{\lambda}_l \omega_l) + \sum_{q=1}^{Q} \mathbf{P}\Delta(\tilde{\boldsymbol{\lambda}}_q \tilde{\omega}_q). \tag{S3}$$

Note that the values $\Delta\tilde{\omega}_q$ may need to shift in order to accommodate any shifts in $\Delta\omega_l$, since the model constrains $\sum_{l=1}^{d_0} \Delta\omega_l + \sum_{q=1}^{Q} \Delta\tilde{\omega}_q = 0$. The first term on the right hand side of (S3) is the target quantity, identifying the desired mixture weights. The second term will be negligible if all profiles $\tilde{\boldsymbol{\mu}}_q$ are approximately orthogonal to the columns of $\mathbf{B}_0$, or else the differences $\Delta\tilde{\omega}_q$ are all small. This condition will be satisfied if S_0 is exhaustive in the sense that $1 - \sum_{l=1}^{d_0} \omega_l^{(z)}$ is negligible.

Mathematically, it is difficult to further characterize the latter two terms, without specifying what *kinds* of non-cell-mediated processes are likely. For example, even if $\Delta\tilde{\boldsymbol{\lambda}}_q = 0$ for a particular value of q, it may nevertheless still produce a bias if $\Delta\tilde{\omega}_q \neq 0$. Conversely, even if $\Delta\omega_l = 0$, bias can result from a nonzero difference $\Delta\boldsymbol{\lambda}_l$ (e.g. different methylation intensities at island shores due to distinct risk profiles) if $\Delta\boldsymbol{\lambda}_l$ is not annihilated by $\mathbf{P}$. Only processes that are equal in intensity in both cases and controls and across all cell types will be differenced out of (S3). Thus, a key consideration is whether $\mathbf{P}$ annihilates the methylation signature corresponding to a given non-cell-mediated biological process. In order to examine this issue more carefully, we adopt a Bayesian view, attempting to characterize a prior expectation of bias as a function of prior probabilities for individual CpG sites. Our goal, in part, is to understand the potential for bias, given the number m of CpG sites chosen to be measured in S_0, with the goal of selecting m in a manner consistent with minimizing bias.

Assume that the CpGs under consideration are ordered in advance (e.g. randomly or by F-statistic $F_j = d_0^{-1}\widehat{\mathbf{B}}_{0j\cdot}\boldsymbol{\Omega}_j^{-1}\widehat{\mathbf{B}}_{0j\cdot}^{\mathrm{T}}$, and explicitly write the dependence of $\mathrm{tr}\mathbf{H}_m = \tilde{\mathbf{B}}_0^{\mathrm{T}}\tilde{\mathbf{B}}_0$ on m. If the CpGs are randomly ordered, then $\mathrm{tr}\mathbf{H}_m = O(m)$, but otherwise it is possible that $\mathrm{tr}\mathbf{H}_m = O(m^{1-\zeta})$, $\zeta > 0$, reflecting a diminishing rate of return by adding additional non-informative CpG sites. Now we decompose $\boldsymbol{\delta} = \sum_{l=1}^{d_0} \mathbf{P}\Delta(\boldsymbol{\lambda}_l\omega_l) + \sum_{q=1}^{Q} \mathbf{P}\Delta(\tilde{\boldsymbol{\lambda}}_q\tilde{\omega}_q)$ by the number k of CpG sites affected by all alterations that distinguish cases from controls. Fix $k \in \mathbb{J}_m = \{1, ..., m\}$; each of the $C(m,k) = m!/[k!(m-k)!]$ subsets $\mathbb{J}_{kl} \subset \mathbb{J}_m$ of k indices corresponds to a vector $\boldsymbol{\delta}_{kl}$ representing the mean methylation difference between case and control over *all* systematic biological processes that result in changes at the k specific CpG sites represented by the k indices, and only those k CpG sites. Thus $\boldsymbol{\delta}_{kl}$ has at most k nonzero values. The bias resulting from such processes is $\mathbf{H}_m^{-1}\tilde{\mathbf{B}}_0^{\mathrm{T}}\boldsymbol{\delta}_{kl} = O(km^{\zeta-1})$. We assume a prior probability π_{kl} that the subset $\mathbb{J}_{kl}$ could correspond to one or more biological processes that distinguish cases from controls. It follows from this view that the prior expectation of $\boldsymbol{\delta}$ is

$$\mathrm{E}[\boldsymbol{\delta}|(\pi_{kl})_{kl}] = \sum_{k=1}^{m} \sum_{l=1}^{C(m,k)} \pi_{kl}\boldsymbol{\delta}_{kl} = O\left(\sum_{k=1}^{m} \sum_{l=1}^{C(m,k)} \pi_{kl} k m^{\zeta-1}\right). \tag{S4}$$

If a prior probability over all sets of CpG sites in the genome is constructed so that CpG sites are considered independent, and each CpG site is assigned a uniform prior probability of π_0, then $\pi_{kl} \equiv \pi_0^k(1-\pi_0)^{m-k}$ and, from (S4),

$$\mathrm{E}(\boldsymbol{\delta}|\pi_0) = O\left(m^{\zeta} \sum_{k=1}^{m} C(m-1, k-1)\pi_0^k(1-\pi_0)^{m-k}\right) = \pi_0(1-\pi_0)O(m^{\zeta}). \tag{S5}$$

It is interesting to note that the bias does not depend on m if $\mathrm{tr}\mathbf{H}_m = O(m)$, i.e. random ordering. However, while random ordering renders the size of $\mathrm{E}(\boldsymbol{\delta}|\pi_0)$ theoretically independent of m, it does so

at the cost of including many potentially noninformative CpGs, early on at low values of m, and these may be possible sources of bias in practice, without offering any modeling benefit in return. If the CpG sites are ordered by level of informativeness, then potentially $\mathbf{H}_m = O(m^{1-\zeta})$, and there will be a small increasing prior expectation of bias, motivating judicious choice of m. The key, then, is to order the CpGs in terms of their ability to distinguish different types profiled in S_0, choosing m large enough to distinguish all signatures from one another, but small enough that the $\mathrm{E}(\boldsymbol{\delta}|\pi_0)$ is reasonably low, in a relative sense. Naturally, different choices of prior π_{kl} in (S4) will lead to different conclusions about the magnitude of bias. If the set $\mathbb{J}_m$ of CpG sites used in S_0 and S_1 oversample those known to have less modifiable methylation states, e.g. away from so-called shore regions [1], then π_0 is effectively lowered, and so will be the corresponding expected prior bias. It is worth emphasizing that this analysis concerns only a Bayesian prior, not the actual biological truth. In our choice of CpG sites among those assayed in S_0 and S_1, we might end up unlucky enough to have included a number of sites that also happen to represent systematic, non-cell-mediated biological differences between cases and controls in S_1, in which case biased estimates will be inevitable. In summary, however, we can exert some control over bias in the proposed estimation procedure by selecting a sufficiently exhaustive list of cell types to profile in S_0, and by choosing m judiciously.

S2.2 Application: Analysis of Sensitivity to Bias

While the bias estimates evident from the double-bootstrap procedure admit the possibility of correcting the bias arising from measurement error, there is no statistical procedure for correcting the other possible sources of bias, those arising from unprofiled cell types and non-cell-mediated profile differences, i.e. methylation difference signatures $\boldsymbol{\delta}$ with nonzero projection onto the space spanned by the WBC signatures. However, it is possible to conduct a sensitivity analysis using the theory presented above. Here, we describe such an analysis for the HNSCC data set presented in the main text.

For each value of $k \in \mathbb{J}_m$, we randomly sampled k elements, $\mathbb{J}_k^* \subset \mathbb{J}_m$ without replacement, then sampled k rows of $\widehat{\mathbf{B}}_1$ without replacement, set $\boldsymbol{\delta}^*$ equal to the $m \times d_1$ zero matrix, and finally substituted the rows indicated by $\mathbb{J}_k^*$ by the k rows selected from $\widehat{\mathbf{B}}_1$. The matrix $\boldsymbol{\delta}^*$ served as a representative of the sum of processes having systematic methylation changes at k locations, of total magnitude consistent with the observed data (under the conservative assumption that *no* systematic methylation difference is cell mediated), and $\boldsymbol{\alpha}^* = (\tilde{\mathbf{B}}_0^{\mathrm{T}}\tilde{\mathbf{B}}_0)^{-1}\tilde{\mathbf{B}}_0^{\mathrm{T}}\boldsymbol{\delta}^*$ represented the corresponding bias in $\widehat{\boldsymbol{\Gamma}}$. If, as in this situation, we were interested in assessing the sensitivity to bias in column of $\mathbf{B}_1$ (i.e. Case Status), we could simply delete the uninteresting columns of $\boldsymbol{\delta}^*$ or $\boldsymbol{\alpha}^*$. Replicating this resampling procedure $100,000$ times, we generated an approximation to the distribution of possible biases corresponding to processes involving exactly k CpG sites.

Figure S1 displays the results of such an analysis, showing the distribution of $({\boldsymbol{\alpha}^*}^{\mathrm{T}}\boldsymbol{\alpha}^*)^{-1/2}$ for various values of k. Note that the relationship of median values to m was consistent with the theory presented in Section 3.3. In fact, the median values of $({\boldsymbol{\alpha}^*}^{\mathrm{T}}\boldsymbol{\alpha}^*)$ had an almost perfect linear relationship with m (data not shown). The magnitude of the bias was small: for the more likely low values of k, the bias was 0.1 to 0.25 of a percentage point. In addition, this analysis was conseravative in that it assumed all of the effect in $\mathbf{B}_1$ was due to non-cell-mediated processes, a strongly conservative assumption.

In addition, for various choices of π_0 over a range of small magnitudes, we computed via iterated expectation the expected bias over the uniform posterior implied by π_0, first by computing the mean bias for each choice of k, then forming the expectation over the binomial distribution $Bin(100, \pi_0)$. As suggested in the previous appendix section, the result scaled linearly with π_0. The constant of proportionality was estimated to be 2.08 percentage points. In summary, if the prior expectation is of even moderate size (~ 0.1) that any one CpG among the 100 selected for this application will show systematic differentiation between cases and controls, then the implied bias would be expected to be less than a percentage point.

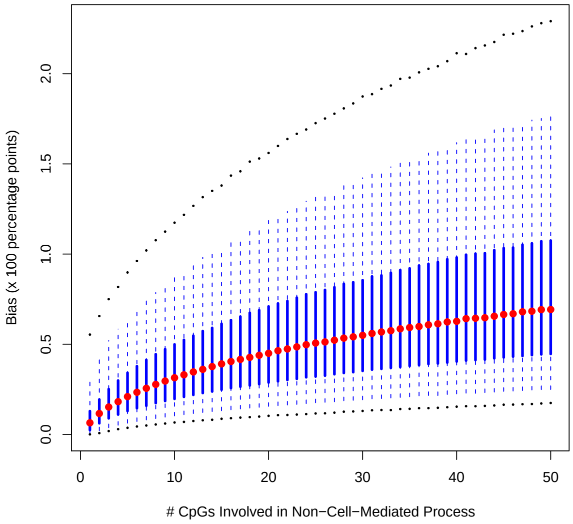

Figure S1. Bias Sensitivity Analysis for HNSCC. Bias assed by resampling the case coefficients of $\mathbf{B}_1$, an procedure that assumes maximum bias. The x-axis show the number of assumed nonzero alterations. The red dots indicate median, the thick blue line indicates interquartile range, the thin lines represent 95% probability ranges, and the outer dots represent 99% probability ranges.

S2.3 Aging and T Cell Methylation

At certain CpG loci, DNA methylation is known to change with age [2], especially in T cells [3]. Consequently, age-related changes in DNA methylation could be another potential source of bias. Figure S2 shows DNA methylation by age in the top 100 CpGs selected for analysis (with a box-plot showing the age-specific distributions and a line-plot showing DNA methylation by age with data from a single CpG connected by a line). The figure suggests that variability in methylation across CpGs is greater than that contributed by age. The clustering heatmap shown in Figure S3, based on z-scores computed by CpG (row), suggests a similar conclusion. The Spearman correlation between age and specimen-specific mean methylation (across all 100 CpGs) was 0.07, with p = 0.74 for the corresponding linear regression coefficient. In addition, we used a recursively partitioned mixture model (RPMM) [4] to cluster the 100 CpGs; no associations were found between age and the four classes determined by RPMM (ANOVA p = 0.24, Kruskal-Wallis p = 0.16). Thus we conclude that any age-related associations with DNA methylation in the top 100 CpGs were too weak to be detected within the current validation sample.

S3 Additional Details Regarding Simulations

S3.1 Additional Notes

The design of our simulations is described in the main paper. However, some additional notes pertain to the details regarding bias provided in this Supplement. In particular, note that the individual, Dirichlet-generated subject weights did not necessarily sum to one, but the difference from 1 was not applied as a multiplier; thus the resulting $\boldsymbol{\xi}$ corresponded to the situation $\mathbf{P}\tilde{\boldsymbol{\mu}}_q = \mathbf{0}$, where $\mathbf{P} \tilde{=} (\tilde{\mathbf{B}}_0^{\mathrm{T}}\tilde{\mathbf{B}}_0)^{-1}\tilde{\mathbf{B}}_0^{\mathrm{T}}$, along with orthogonal contributions from the λ terms of (S2).

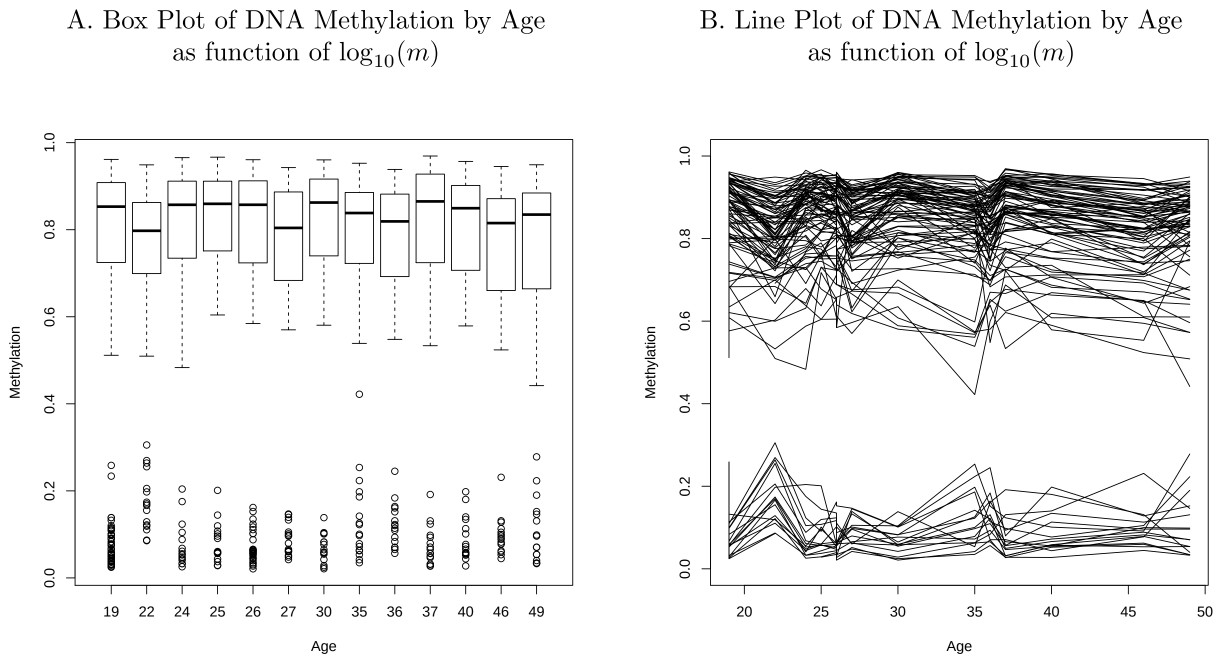

Figure S2. DNA methylation by Age in T Cells H_m.

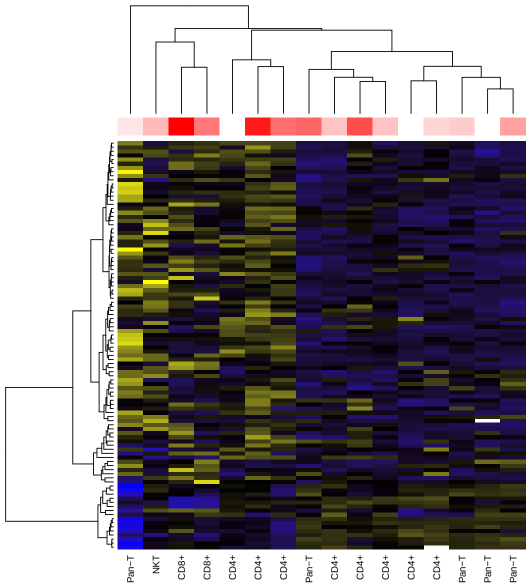

Figure S3. Clustering Heatmap for External Validation T Cells, Standardized and Annotated with Age. Yellow = relatively unmethylated (row z score << 0), black = partially methylated (row z score ≈ 0), blue = relatively methylated (row z score >> 0). The annotation track above the heatmap indicates age status (white = younger, red = older).

S3.2 Results for Coefficients of Determination

Complete results for the coefficients of determination are provided in Table S1. $R^2_{1,0}$ decreased with decreasing strength of the alternative, falling to zero under both null scenarios. For strong alternatives, $R^2_{1,1}$ was frequently close to 1.0. For the Mixed Alternative, $R^2_{1,1}$ had a lower, but still high values ranging from about 0.85 to 0.90. For the Mixed Null result, $R^2_{1,1}$ typically had lower values, from about 0.05 to 0.20. In the Strong Null case, $R^2_{1,1}$ covered a broader range among moderately low values; note, however, that this scenario effectively represents 0/0, i.e. a poorly defined value.

Table S1. Results for Coefficients of Determination

		Median $R^2_{1,0}$ (Interquartile Range)	Median $R^2_{1,1}$ (Interquartile Range)
Precise Mixtures	Strong Alternative I ($\theta = 0$)	0.13 (0.12-0.15)	0.98 (0.97-0.98)
$n_1 = 200$	Strong Alternative II ($\theta = 0$)	0.13 (0.12-0.15)	0.98 (0.97-0.98)
	Mixed Alternative ($\theta = 0.5$)	0.04 (0.03-0.05)	0.88 (0.85-0.91)
	Mixed Null ($\theta = 1$)	0.00 (0.00-0.00)	0.10 (0.05-0.17)
	Strong Null ($\theta = 0$)	0.00 (0.00-0.00)	0.25 (0.15-0.38)
Noisy Mixtures	Strong Alternative I ($\theta = 0$)	0.05 (0.03-0.06)	0.98 (0.97-0.98)
$n_1 = 200$	Strong Alternative II ($\theta = 0$)	0.05 (0.03-0.06)	0.98 (0.97-0.98)
	Mixed Alternative ($\theta = 0.5$)	0.01 (0.01-0.02)	0.89 (0.81-0.94)
	Mixed Null ($\theta = 1$)	0.00 (0.00-0.01)	0.46 (0.28-0.64)
	Strong Null ($\theta = 0$)	0.00 (0.00-0.01)	0.72 (0.55-0.85)

S3.3 Additional Simulations

We conducted additional simulations (details not show), which assumed bias arising from processes not profiled by the profiled leukocytes. For these scenarios, $\boldsymbol{\xi}^0$ was set to $\widehat{\boldsymbol{\mu}}_1$, and $\boldsymbol{\xi}^1 = \boldsymbol{\xi}^0$ except for a set of CpG sites randomly selected among the m dimensions of the array (once and for all before all 1000 simulations); among those dimensions j, $\boldsymbol{\xi}^1_j$ was set to $1 - \widehat{\boldsymbol{\mu}}_{1j}$, reflecting a "reversal" of methylation state. Estimates were biased towards the null, on the order of about a percentage point

S4 Supplement to Results

S4.1 Selection of CpG Sites

From S_0, F statistics $F_j = 6^{-1}\mathbf{L}^{\mathrm{T}}\widehat{\mathbf{B}}_{0j\cdot}\boldsymbol{\Omega}_j^{-1}\widehat{\mathbf{B}}_{0j\cdot}^{\mathrm{T}}\mathbf{L}$, were computed and used to order each of the 26,486 autosomal CpGs by decreasing level of informativeness with respect to blood cell types. Figure S4A depicts the relationship $\log_{10}\mathrm{tr}\mathbf{H}_m$ by $\log_{10}(m)$ for increasing array sizes. Figure S4B depicts the relationship $\partial \log_{10}\mathrm{tr}(\mathbf{H}_m)/\partial \log(m)$ by $\log_{10}(m)$ for increasing array sizes, obtained by smoothing the first-differences of the curve depicted in Figure S4A via loess smoother. Figure S4A also shows the tangent (obtained from the loess curve) at low values of m. For $O(m)$ convergence, Figure S4A should show a linear association with slope equal to one, while the curve in Figure S4B should show a curve close to the value of 1.0. Neither is the case, i.e. convergence is sub-linear in m. Note that the rate of convergence dropped precipitously after about 6,000 CpG sites, but was notably slower than $O(m)$ even after $m = 10$. In the range of 1-1000 CpG sites the convergence rate appeared parabolic with a minimum of about 0.85, starting to stabilize in the $m = 100 - 300$ range.

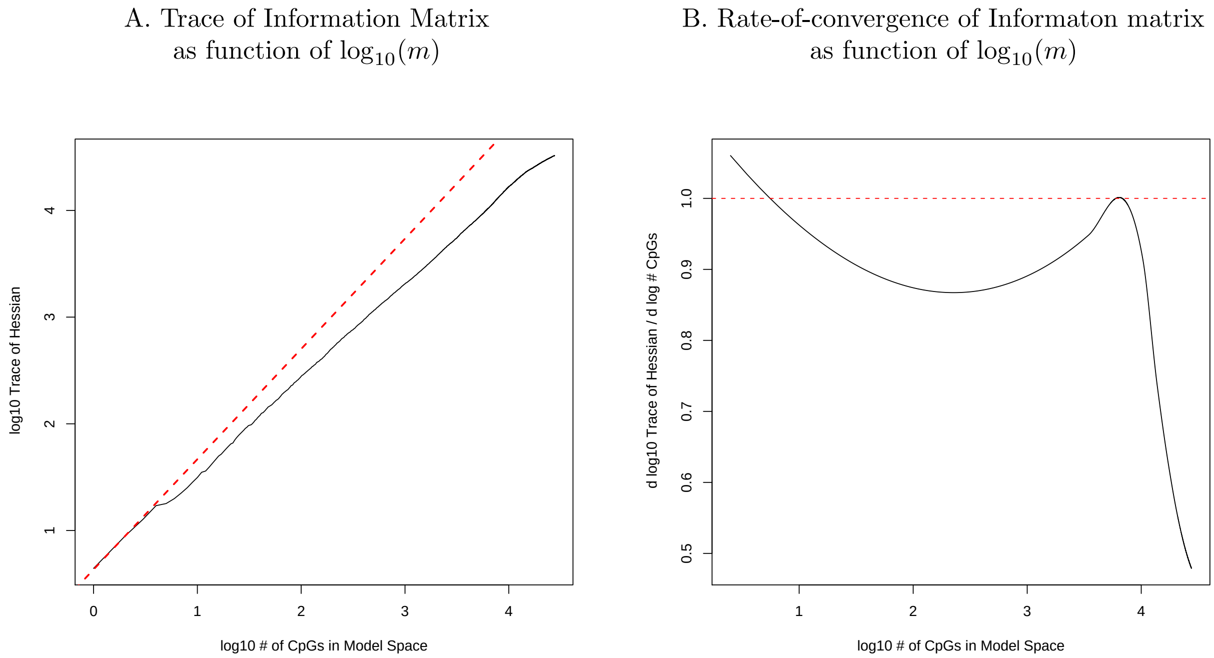

Figure S4. Rate-of-Convergence for $\mathbf{H}_m$. The x-axis represents increasing m, the number of CpG sites (ordered by F statistic) included in the model-space, on a logarithmic scale. Rate-of-convergence was calculated by smoothing the first differences of $\log_{10}(\text{tr}\mathbf{H}_m)$. The dotted red line in (A) shows the tangent at low values of m, while the dotted red line in (B) corresponds to linear convergence.

S4.2 Additional Clustering Heatmaps

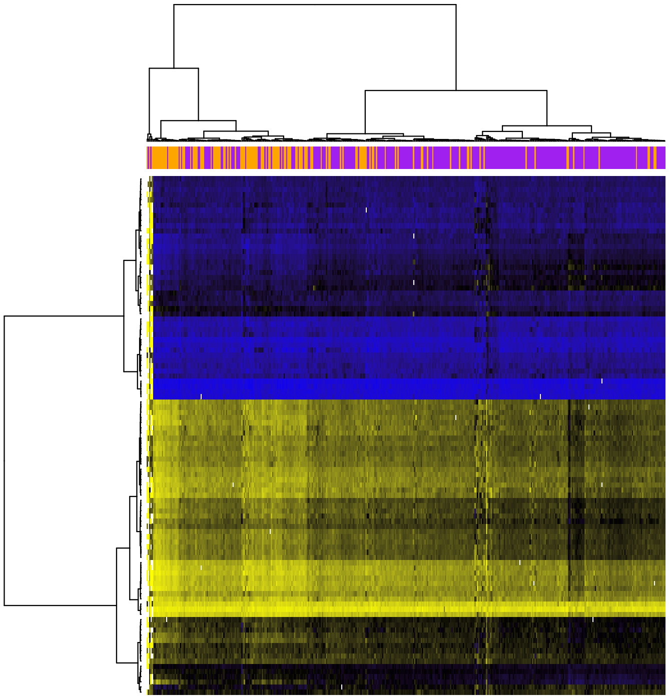

Figure S5. Clustering Heatmap for Target Ovarian Cancer Data. Yellow = unmethylated ($Y_{ij} = 0$), black = partially methylated ($Y_{ij} = 0.5$), blue = methylated ($Y_{ij} = 1$). The annotation track above the heatmap indicates case-control status (orange = cancer case, purple = control).

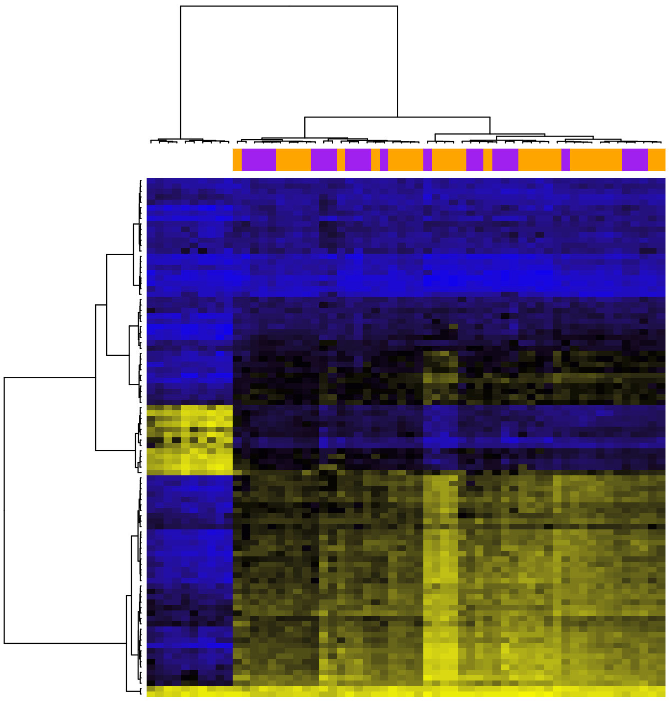

Figure S6. Clustering Heatmap for Target Down Syndrome Data. Yellow = unmethylated ($Y_{ij} = 0$), black = partially methylated ($Y_{ij} = 0.5$), blue = methylated ($Y_{ij} = 1$). The annotation track above the heatmap indicates case-control and cell type status [orange = Down syndrome case (whole blood), purple = control (whole blood), white=T-cell (pooled cases and controls)].

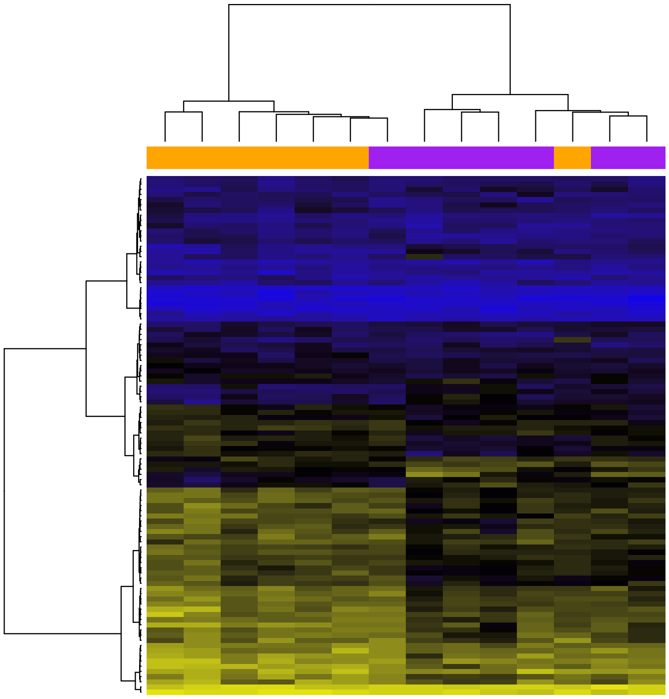

Figure S7. Clustering Heatmap for Target Obesity Data. Yellow = unmethylated ($Y_{ij} = 0$), black = partially methylated ($Y_{ij} = 0.5$), blue = methylated ($Y_{ij} = 1$). The annotation track above the heatmap indicates case-control status (orange = obese, purple = lean).

S4.3 Additional Regression Coefficient Estimates

Table S2. Estimated Regression Coefficients for Sex and Age in HNSCC Data Set

		Est	$Bias_2$	SE_0	SE_1	SE_2	P-value
Sex	(Intercept, γ_0)	0.12	0.00	0.24	0.57	0.57	0.83
	B Cell	0.38	0.01	0.17	0.85	0.84	0.65
	Granulocyte	-0.29	-0.08	0.28	1.82	1.81	0.87
	Monocyte	0.13	0.01	0.29	0.47	0.47	0.78
	NK	0.49	0.05	0.32	0.40	0.40	0.22
	T Cell (cd4+)	-1.80	0.45	1.12	1.25	1.20	0.13
	T Cell (cd8+)	0.82	-0.44	1.12	1.03	1.04	0.43
(Age - 60)/10	(Intercept, γ_0)	-0.20	-0.02	0.15	0.24	0.24	0.40
	B Cell	0.24	0.01	0.11	0.34	0.33	0.47
	Granulocyte	1.12	-0.01	0.19	0.67	0.67	0.096
	Monocyte	0.13	0.02	0.19	0.20	0.20	0.54
	NK	-0.22	0.02	0.21	0.15	0.15	0.14
	T Cell (cd4+)	-2.75	0.56	0.73	0.53	0.57	<0.0001
	T Cell (cd8+)	1.44	-0.56	0.73	0.46	0.50	0.0038

Est = Regression coefficient estimate ($\times$ 100%)
$Bias_2$ = Double-bootstrap bias estimate ($\times$ 100%).
SE_0 = Naive standard error ($\times$ 100%).
SE_1 = Single-bootstrap standard error ($\times$ 100%).
SE_2 = Double-bootstrap standard error ($\times$ 100%).
P-values were computed using SE_2.

Table S3. Estimated Regression Coefficients for Age in Ovarian Cancer Data Set

		Est	$Bias_2$	SE_0	SE_1	SE_2	P-value
Age 55-60	(Intercept, γ_0)	-1.24	-0.05	0.37	0.41	0.40	0.0021
	B Cell	0.40	0.04	0.27	0.50	0.49	0.42
	Granulocyte	0.91	0.04	0.45	2.04	2.02	0.65
	Monocyte	0.85	0.12	0.45	0.59	0.58	0.15
	NK	-0.25	0.10	0.50	0.55	0.55	0.65
	T Cell (cd4+)	-2.79	0.63	1.76	2.13	1.96	0.15
	T Cell (cd8+)	2.22	-0.84	1.77	1.81	1.59	0.16
Age 60-65	(Intercept, γ_0)	-0.72	-0.07	0.35	0.39	0.39	0.070
	B Cell	0.54	0.07	0.25	0.49	0.49	0.27
	Granulocyte	0.71	0.06	0.42	1.99	1.98	0.72
	Monocyte	0.27	0.08	0.42	0.58	0.58	0.64
	NK	-0.24	0.06	0.47	0.55	0.55	0.65
	T Cell (cd4+)	-3.54	0.80	1.66	2.02	1.97	0.072
	T Cell (cd8+)	2.84	-0.97	1.66	1.85	1.64	0.084
Age 65-70	(Intercept, γ_0)	-0.53	-0.08	0.40	0.41	0.41	0.19
	B Cell	-0.03	0.07	0.29	0.51	0.51	0.96
	Granulocyte	2.46	0.02	0.48	2.17	2.17	0.26
	Monocyte	0.85	0.12	0.48	0.64	0.64	0.18
	NK	-0.89	0.07	0.54	0.59	0.60	0.14
	T Cell (cd4+)	-6.12	1.48	1.89	2.18	2.12	0.0038
	T Cell (cd8+)	4.37	-1.64	1.89	1.87	1.71	0.011
Age 70-75	(Intercept, γ_0)	-1.20	-0.07	0.40	0.41	0.41	0.0037
	B Cell	0.29	0.07	0.29	0.48	0.48	0.55
	Granulocyte	2.13	-0.05	0.48	2.05	2.04	0.30
	Monocyte	0.76	0.12	0.48	0.60	0.60	0.21
	NK	-0.51	0.19	0.54	0.56	0.55	0.36
	T Cell (cd4+)	-6.82	1.97	1.89	2.16	2.12	0.0013
	T Cell (cd8+)	5.35	-2.20	1.90	1.89	1.79	0.0028
Age 75+	(Intercept, γ_0)	-0.31	-0.09	0.49	0.46	0.45	0.49
	B Cell	0.13	0.08	0.35	0.54	0.53	0.81
	Granulocyte	1.10	-0.15	0.58	2.12	2.11	0.60
	Monocyte	1.73	0.12	0.59	0.64	0.63	0.0065
	NK	-0.30	0.13	0.66	0.60	0.59	0.61
	T Cell (cd4+)	-6.54	1.31	2.30	2.29	2.18	0.0027
	T Cell (cd8+)	2.73	-1.37	2.31	2.06	1.86	0.14

Est = Regression coefficient estimate (× 100%)
$Bias_2$ = Double-bootstrap bias estimate (× 100%).
SE_0 = Naive standard error (× 100%).
SE_1 = Single-bootstrap standard error (× 100%).
SE_2 = Double-bootstrap standard error (× 100%).
P-values were computed using SE_2.

Table S4. Estimated Regression Coefficients for Bisulfite Conversion in Ovarian Cancer Data Set

		Est	$Bias_2$	SE_0	SE_1	SE_2	P-value
BSC1	(Intercept, γ_0)	-0.08	0.00	0.14	0.09	0.10	0.39
(Green	B Cell	-0.10	0.00	0.10	0.10	0.10	0.30
/1000)	Granulocyte	0.13	0.04	0.17	0.40	0.40	0.74
	Monocyte	0.13	-0.01	0.17	0.12	0.12	0.26
	NK	-0.09	0.00	0.19	0.14	0.14	0.53
	T Cell (cd4+)	0.51	-0.14	0.65	0.48	0.51	0.32
	T Cell (cd8+)	-0.23	0.11	0.66	0.40	0.47	0.62
BSC2	(Intercept, γ_0)	0.25	0.00	0.14	0.08	0.08	0.0027
(Green	B Cell	0.07	0.00	0.10	0.08	0.08	0.40
/1000)	Granulocyte	0.07	0.01	0.17	0.38	0.37	0.84
	Monocyte	-0.18	0.01	0.17	0.10	0.10	0.075
	NK	0.10	0.00	0.19	0.12	0.12	0.41
	T Cell (cd4+)	-0.65	0.20	0.67	0.41	0.50	0.20
	T Cell (cd8+)	0.63	-0.21	0.68	0.34	0.45	0.16

Est = Regression coefficient estimate ($\times$ 100%)
$Bias_2$ = Double-bootstrap bias estimate ($\times$ 100%).
SE_0 = Naive standard error ($\times$ 100%).
SE_1 = Single-bootstrap standard error ($\times$ 100%).
SE_2 = Double-bootstrap standard error ($\times$ 100%).
P-values were computed using SE_2.
Note that coefficients are given as % / 1000 units fluorescence, and that standard deviations for BSC1 and BSC2 were 1950 and 2169, respectively.

Table S5. Estimates for Down Syndrome Analysis (Case vs. Control, Total Leukocyte vs. T Cell)

		Est	$Bias_2$	SE_0	SE_1	SE_2	P-value
Case Status	(Intercept, γ_0)	2.02	-0.10	0.86	1.17	1.17	0.084
(total leukocytes)	B Cell	-4.87	-0.03	0.62	0.70	0.69	<0.0001
	Granulocyte	3.85	0.15	1.02	3.01	2.98	0.20
	Monocyte	0.12	0.11	1.03	0.97	0.96	0.90
	NK	-0.63	-0.06	1.16	0.83	0.82	0.44
	T Cell (cd4+)	-0.30	-0.37	4.02	2.49	2.66	0.91
	T Cell (cd8+)	-1.89	0.35	4.03	2.47	2.42	0.43
T Cell	(Intercept, γ_0)	-0.97	0.07	1.7	1.4	1.6	0.54
(cases+controls)	B Cell	-0.51	0.02	1.2	1.2	1.2	0.67
	Granulocyte	-56.21	0.49	2.1	3.4	3.4	<0.0001
	Monocyte	-5.13	-0.37	2.1	1.1	1.3	<0.0001
	NK	0.07	0.34	2.3	1.5	1.7	0.97
	T Cell (cd4+)	60.18	-2.89	8.1	3.2	5.2	<0.0001
	T Cell (cd8+)	3.00	2.34	8.2	3.3	5.4	0.58

Est = Regression coefficient estimate ($\times$ 100%).
$Bias_2$ = Double-bootstrap bias estimate ($\times$ 100%).
SE_0 = Naive standard error ($\times$ 100%).
SE_1 = Single-bootstrap standard error ($\times$ 100%).
SE_2 = Double-bootstrap standard error ($\times$ 100%).
P-values were computed using SE_2.

Table S6. Estimated Regression Coefficients for Data Set concerning Obesity in African Americans

		Est	$Bias_2$	SE_0	SE_1	SE_2	P-value
Obese	(Intercept, γ_0)	0.96	-0.09	1.08	0.85	0.84	0.25
	B Cell	0.70	-0.03	0.78	1.16	1.14	0.54
	Granulocyte	12.25	0.51	1.30	4.27	4.27	0.0041
	Monocyte	-0.70	-0.01	1.31	1.57	1.54	0.65
	NK	-4.42	-0.13	1.46	1.75	1.73	0.011
	T Cell (cd4+)	-6.97	-0.29	5.11	6.27	5.49	0.20
	T Cell (cd8+)	-2.29	0.22	5.13	4.97	4.36	0.60

Est = Regression coefficient estimate ($\times$ 100%).
$Bias_2$ = Double-bootstrap bias estimate ($\times$ 100%).
SE_0 = Naive standard error ($\times$ 100%).
SE_1 = Single-bootstrap standard error ($\times$ 100%).
SE_2 = Double-bootstrap standard error ($\times$ 100%).
P-values were computed using SE_2.

S5 Analysis of Intercepts

If the subject population for which $\mathbf{z} = \mathbf{0}$ is sufficiently homogeneous with respect to blood cell distribution to admit sensible characterization of that distribution, then it is possible to recover estimates from $\widehat{\mathbf{\Gamma}}$. Table S7 displays the results of such an analysis applied to the HNSCC case/control data set. If the coefficients represented a complete profiling of blood cell types, the estimates should sum approximately to one, even though the model does not explicitly constrain them so. In this case, the original bias-corrected estimates (of leukocyte distribution in HNSCC controls) summed to 133%. The table shows the values re-normalized to 90%, the anticipated proportion of the cell types. The resulting estimated distribution of leukocytes is consistent with the literature [5].

Table S7. White Blood Cell Distribution in HNSCC Controls

	Est	SE_2	$Bias_2$	BC-Est	95% Conf. Int.
B-Cell	7.9	0.5	0.1	7.8	$(6.8, 8.9)$
Granulocyte	42.2	1.2	-0.1	42.3	$(39.9, 44.6)$
Monocyte	9.9	0.7	0.3	9.6	$(8.3, 10.9)$
NK	7.9	0.7	0.2	7.7	$(6.3, 9.1)$
T Cell (cd4+)	15.2	3.0	-0.1	15.3	$(9.5, 21.2)$
T Cell (cd8+)	7.6	3.0	0.4	7.2	$(1.4, 13.0)$

Est = Regression coefficient estimate ($\times$ 100%), normalized so that estimates sum to 90%.
SE_2 = Double-bootstrap standard error ($\times$ 100%).
$Bias_2$ = Double-bootstrap bias estimate ($\times$ 100%).
BC-Est = bias-corrected estimate.

S6 Application of Proposed Methodology to mRNA Data

Numerous authors have proposed immunological explanations for differences in mRNA profiles between cases and controls, e.g. Showe et al. (2009) [6] and Kossenkov et al. (2011) [7]. The statistical principles described in this article would apply, wholesale, to mRNA expression profiles, but with two cautionary statements. The first is mathematical: mRNA is typically analyzed on a logarithmic scale, yet the assumptions of the proposed methodology involve linearity on an arithmetic scale, since the mixing coefficients are assumed to act linearly on absolute numbers of nucleic acid molecules; thus, the proposed methods would require analysis of untransformed fluorescence intensities, whose skewed distributions would result in numerical instabilities. The second is biological: there is no necessarily linear relationship between cell number and mRNA copies, since proteins may be translated as a consequence of an initial burst of mRNA transcription upon cellular development, after which significant mRNA degradation is possible. In contrast, one would expect the average beta value provided by Illumina bead-array products, as well as similarly constructed quantities from other platforms, to scale in proportion to the actual fraction of methylated nucleic acids; in addition, an assumption of two DNA molecules per cell seems biologically reasonable.

Nevertheless, we provide an example of an application of our methods using mRNA data. The validation data set S_0 was obtained from Ref. [8], who employed the Illumina Human-6 v2 Expression BeadChip to characterize the mRNA expresion profile of 8 types of blood cells: B cells, granulocytes, erythroblasts, megakaryocytes, monocytes, natural killer cells, cd4+ T cells, and cd8+ T cells. For this analysis we removed erythroblasts (nucleated progenitors of red blood cells) and megakaryocytes (progenitors of platelets). The target data set S_1 was obtained from Ref. [6], who used the same mRNA expression platform to characterize expression differences in isolated mononuclear cells between nonsmall cell lung cancer (NSCLC) cases and controls having non-cancer lung disease, adjusting for age, sex and smoking. In addition, they present data from 18 matched case samples, pre- and post-operative.

We employed the same methodology as for the DNA methylation data sets, ordering the 46,693 transcripts by F statistic according to their ability to distinguish six types of leukocytes. 86 of the 100 transcripts having the largest F statistics overlapped with the transcripts made available by Ref. [6]. Thus we applied the remainder of the analysis using the 86 overlapping loci. In all analyses, we used untransformed data (i.e. using either the normalized fluorescence intensities or 2 raised to the power of the normalized $\log_2$ intensities). Application of the constrained projection proposed in Section 2 of the main article resulted in an average percentage estimates somewhat consistent with mononuclear cells (i.e. a subfraction with most granulocytes removed): 3.3% B cell, 3.4% granulocyte, 18.1% monocyte, 29.5 % NK cell, 11.6 CD4+ T cell, and 2.2 % CD8+ T cell.

Table S8 presents results from 137 NSCLC cases and 91 controls, adjusted for age, sex, and smoking status. Table S9 presents results from 18 matched pre-operative and post-operative samples from NSCLC cases, where the analyzed outcome was the difference in untransformed expression (post-operative expression minus pre-operative expression), and coefficients displayed correspond to the intercept of $\mathbf{B}_1$ (analogous to a paired t-test). Perturbations in T-cell distribution are consistent with known immunological changes resulting from NSCLC [9, 10], as well as age and smoking. While the perturbations and coefficient signs seem reasonable, the magnitudes are potentially quite biased. For example, the estimates corresponding to granulocyte distribution are much larger than one would expect given the relatively small number of granulocytes present in a monouclear subfraction. Thus, while it is possible to employ our proposed methodology on mRNA data sets, we are much less confident that the method will produce reliable results, given the physical and mathematical limitations of mRNA compared with DNA methylation.

Table S8. White Blood Cell Distribution Comparing Cases to Controls in HNSCC mRNA Data Set

	Est	SE_2	p-value
Case Status			
B Cell	0.8	4.15	0.8511
Granulocyte	-34.6	9.48	0.0003
Monocyte	17.9	9.58	0.0613
NK	1.3	5.18	0.8095
T Cell (cd4+)	24.9	9.01	0.0057
T Cell (cd8+)	-15.2	9.03	0.0931
Age (decades)			
B Cell	-0.7	1.36	0.5824
Granulocyte	-7.9	3.45	0.0218
Monocyte	-6.5	2.76	0.0180
NK	-4.0	1.80	0.0255
T Cell (cd4+)	13.0	2.89	0.0000
T Cell (cd8+)	8.3	2.96	0.0052
Sex (male)			
B Cell	0.1	2.66	0.9827
Granulocyte	-34.8	6.41	0.0000
Monocyte	6.8	5.44	0.2091
NK	-7.8	3.32	0.0193
T Cell (cd4+)	21.1	5.39	0.0001
T Cell (cd8+)	13.2	5.76	0.0223
Former Smoker			
B Cell	1.6	3.97	0.6821
Granulocyte	17.2	8.25	0.0375
Monocyte	6.1	7.84	0.4368
NK	2.7	5.19	0.6103
T Cell (cd4+)	-11.3	8.02	0.1578
T Cell (cd8+)	-20.3	8.28	0.0141
Current Smoker			
B Cell	3.4	5.21	0.5183
Granulocyte	31.6	11.26	0.0049
Monocyte	17.8	10.49	0.0907
NK	5.4	6.93	0.4373
T Cell (cd4+)	-21.8	10.25	0.0337
T Cell (cd8+)	-41.2	11.10	0.0002

Est = Regression coefficient estimate ($\times$ 100%)
SE_2 = Double-bootstrap standard error ($\times$ 100%).

Table S9. White Blood Cell Distribution Comparing Matched Pre-operative and Post-operative Cases in HNSCC mRNA Data Set

	Est	SE_2	p-value
B Cell	-10.7	5.55	0.0543
Granulocyte	-19.4	11.16	0.0826
Monocyte	-13.4	10.43	0.1987
NK	6.3	7.15	0.3794
T Cell (cd4+)	-11.3	10.57	0.2859
T Cell (cd8+)	48.8	11.33	0.0000

Est = Regression coefficient estimate (× 100%)
SE_2 = Double-bootstrap standard error (× 100%).

References

1. Doi A, Park IH, Wen B, Murakami P, Aryee MJ, et al. (2009) Differential methylation of tissue- and cancer-specific CpG island shores distinguishes human induced pluripotent stem cells, embryonic stem cells and fibroblasts. Nat Genet 41: 1350-3.

2. Bocklandt S, Lin W, Sehl ME, Sanchez FJ, Sinsheimer JS, et al. (2011) Epigenetic predictor of age. PLoS One 6: e14821.

3. Chu M, Siegmund KD, Hao QL, Crooks GM, Tavare S, et al. (2006) Inferring relative numbers of human leucocyte genome replications. Br J Haematol 141: 862-71.

4. Houseman EA, Christensen BC, Yeh RF, Marsit CJ, Karagas MR, et al. (2008) Model-based clustering of dna methylation array data: a recursive-partitioning algorithm for high-dimensional data arising as a mixture of beta distributions. BMC Bioinformatics 9: 365.

5. Alberts B, Johnson A, Lewis J, Raff M, Roberts K, et al. (2008) Molecular Biology of the Cell. New York, NY: Taylor & Francis, 5th edition.

6. Showe MK, Vachani A, Kossenkov AV, Yousef M, Nichols C, et al. (2009) Gene expression profiles in peripheral blood mononuclear cells can distinguish patients with non-small cell lung cancer from patients with nonmalignant lung disease. Cancer Res 69: 9202-10.

7. Kossenkov AV, Vachani A, Chang C, Nichols C, Billouin S, et al. (2011) Resection of non-small cell lung cancers reverses tumor-induced gene expression changes in the peripheral immune system. Clin Cancer Res 17: 5867-77.

8. Watkins NA, Gusnanto A, de Bono B, De S, Miranda-Saavedra D, et al. (2009) A haematlas: characterizing gene expression in differentiated human blood cells. Blood 113: e1-e9.

9. Ginns LC, Goldenheim PD, Miller LG, Burton RC, Gillick L, et al. (1982) T-lymphocyte subsets in smoking and lung cancer: analysis of monoclonal antibodies and flow cytometry. Am Rev Respir Dis 23: 265–9.

10. Mazzoccoli G, Balzanelli M, Giuliani A, De Cata A, La Viola M, et al. (1999) Lymphocyte subpopulations anomalies in lung cancer patients and relationship to the stage of disease. In Vivo 13: 205–9.
